# Efficacy and safety of different traditional Chinese medicine injections in the treatment of unstable angina pectoris: a systematic review and Bayesian network meta-analysis

**DOI:** 10.3389/fphar.2025.1550759

**Published:** 2025-03-12

**Authors:** Qiuhan Chen, He Wang, Lin Zhu, Ziyi Guo, Yingying Cui, Jifang Ban, Kuo Chi, Na Shi, Boyu Wang, Changxing Liu, Yabin Zhou

**Affiliations:** ^1^ First Clinical Medical School, Heilongjiang University of Chinese Medicine, Harbin, Heilongjiang, China; ^2^ Department of Cardiology, First Hospital of Heilongjiang University of Chinese Medicine, Harbin, Heilongjiang, China; ^3^ Fever Clinic, First Hospital of Heilongjiang University of Chinese Medicine, Harbin, Heilongjiang, China

**Keywords:** traditional Chinese medicine, injection, unstable angina pectoris, Bayesian network meta-analysis, systematic review

## Abstract

**Objectives:**

Several studies have explored the efficacy and safety of various traditional Chinese medicine (TCM) injections for unstable angina pectoris. However, comprehensive systematic evidence confirming the advantages of these injections is still lacking. This Bayesian network meta-analysis was carried out to evaluate and compare the efficacy of different TCM injections in treating unstable angina pectoris.

**Methods:**

A systematic search was implemented across PubMed, Cochrane Library, Embase, and Web of Science, with the date of search cutoff being February 2024. The Cochrane risk of bias tool was utilized to evaluate the bias risk in the included studies.

**Results:**

A total of 44 studies, encompassing 4,362 patients with unstable angina pectoris and 21 types of injections, were included. Compared with the standard treatment group, Danhong injection (SMD = −1.1, 95% CrI: -2.0, −0.15), Danshen Chuanxiongqin injection (SMD = −1.9, 95% CrI: -3.7, −0.12), Ginkgo Damole injection (SMD = −2.5, 95% CrI: -4.8, −0.29), Puerarin injection (SMD = −1.8, 95% CrI: -3.2, −0.37), and Shuxuetong injection (SMD = −7.8, 95% CrI: -13, −2.3) were found to significantly reduce the frequency of angina attacks. However, no significant improvement was observed in the duration of angina episodes with any of the included TCM injections compared with the standard treatment group. There was no significant difference in the incidence of adverse events from TCM injections.

**Conclusion:**

Adjunctive treatment with TCM injections, in addition to conventional therapy, can remarkably reduce the frequency of angina attacks and demonstrates a favorable safety profile. However, it does not appear to significantly reduce the duration of angina episodes. Future studies should include more multicenter populations to validate our conclusions, as the population included in this study was predominantly Chinese.

**Systematic Review Registration:**

identifier [CRD42024501984].

## 1 Introduction

Unstable angina pectoris (UAP), which lies between acute myocardial infarction and stable angina, is a clinical state resulting from acute myocardial ischemia and is categorized under acute coronary syndrome (ACS). One-third of deaths worldwide are due to cardiovascular disease (Roth et al., 2020). The risk factors include age, dyslipidemia, diabetes mellitus, and hypertension. UAP is a complex condition that significantly affects patients’ quality of life. Studies have indicated that approximately 30% of UAP patients progress to myocardial infarction within 3–4 months after onset (Braunwald et al., 2002), with a high mortality.

Currently, treatment regimens for UAP vary widely, focusing on invasive procedures and drug therapy (AuthorAnonymous, 2024). Common invasive procedures for UAP include percutaneous coronary intervention and coronary artery bypass grafting (Bhatt et al., 2022). Drug therapy for UAP includes several classes of medications (Roth et al., 2020): anti-ischemic agents, such as nitrates (for example, nitroglycerin) and β-blockers (for example, metoprolol) (Braunwald et al., 2002); antiplatelet agents, including COX inhibitors (for example, aspirin) and glycoprotein IIb/IIIa receptor antagonists (for example, tirofiban) (AuthorAnonymous, 2024); anticoagulants, such as heparin and low-molecular-weight heparin (Bhatt et al., 2022); lipid-lowering agents, such as statins, which reduce lipids and prevent thrombosis; and (Huang et al., 2024) antihypertensives, including angiotensin-converting enzyme inhibitors and β-blockers. Remote ischemic conditioning has been shown to reduce peak troponin levels and the risk of T4aMI and MACE (Huang et al., 2024). However, despite the effectiveness of biomedical treatments, they are often associated with adverse reactions. For instance, aspirin can cause hypersensitivity reactions (Laidlaw and Cahill, 2017), and in older patients, it increases the overall risk of gastrointestinal hemorrhage by 60% (Mahady et al., 2021). Also, heparin and its derivatives may induce hyperkalemia as a drug-related adverse effect (Ben Salem et al., 2014).

TCM has attracted global attention and has been widely used in clinical practice in recent years. It has demonstrated multi-target, multi-level, and multi-pathway effects in treating coronary artery disease by regulating lipid metabolism, inhibiting inflammatory responses, and protecting myocardial cells. Among oral TCM formulations, Shengshao capsules, Naoxintong capsules, Yinxing Tongzhi dropping pills, and compound Danshen dropping pills have been shown to be effective in treating UAP (Zhang et al., 2021). TCM injections, such as Danhong injection and Danshen injection, are gradually being used for this disease. Moreover, some original studies have indicated that TCM injections offer advantages over standard biomedicine alone. In addition, the therapeutic efficacy of diversified TCM injection treatment regimens remains a subject of debate. Although multiple TCM injections have been revealed to be effective in the treatment of UAP, many questions remain to be studied. For instance, are these injections effective in reducing the frequency of attacks or duration of attacks? Do they effectively reduce both the frequency and duration of attacks, or are they only effective for one of these outcomes? Are they more effective than conventional treatments? Do they carry an increased risk of adverse events? Some systematic reviews (Zhang D. et al., 2016; Ma et al., 2015) have assessed the efficacy and safety of TCM injections in the treatment of UAP. However, given the wide variety of TCM injections used in UAP management, the differences in efficacy among different TCM injections remain to be fully explored. Thus, in order to provide evidence-based recommendations for the clinical usage of TCM injections in the treatment of unstable angina, we carried out this systematic review and Bayesian network meta-analysis to compile the clinical efficacy of these TCM injections.

## 2 Methods

### 2.1 Study registration

This study was implemented per the Preferred Reporting Items for Systematic Reviews and Meta-Analyses for Network Meta-Analyses (PRISMA-NMA) guidelines and was registered with PROSPERO prospectively (ID: CRD42024501984).

### 2.2 Eligibility criteria

#### 2.2.1 Inclusion criteria

P (Population): The target population for this systematic review consisted of patients diagnosed with UAP.

I (Intervention): The intervention for this systematic review included various TCM injections. The information on the components of the traditional Chinese medicine injections discussed in this study can be found in Supplementary Table S1.

C (Comparison): The comparison group consisted of traditional biomedical treatments.

O (Outcome): Frequency and duration of angina attacks.

S (Study design): Randomized controlled trials (RCTs).

#### 2.2.2 Exclusion criteria

Conference abstracts published without peer review;

Studies that only compared different doses or frequencies of the same injection within the original research, as these cannot be linked with other interventions in a Bayesian network meta-analysis.

Studies with very small sample sizes (<10 cases) in the original research, as these are prone to operational errors. Studies with limited subjects are more susceptible to measurement errors. Therefore, these studies with limited subjects should be excluded. In addition, studies with a total of less than 10 subjects in 2 groups had difficulty in meeting statistical efficiency, requiring the exclusion of these studies.

### 2.3 Data sources and search strategy

PubMed, Cochrane Library, Embase, Web of Science, CNKI, Wanfang, VIP, and the Chinese Biomedical Database were all thoroughly searched. The initial search was conducted on 1 November 2023, followed by a supplementary search in February 2024 to minimize the risk of omitting relevant studies. The search strategy utilized a combination of MeSH and free text terms, with no restrictions on region or publication date.

### 2.4 Study selection

Duplicate articles were removed after the retrieved articles were imported into Endnote. After that, titles and abstracts of the remaining research were examined in order to determine which ones were preliminary eligible investigations. Full texts of the remaining were obtained and examined to determine which studies were suitable for inclusion in this systematic review. Two researchers (CQH, WH) carried out the study selection process independently, followed by cross-checking. A third researcher (ZL) was consulted to settle any disagreements.

### 2.5 Data extraction

A standardized data extraction form was created before any data was extracted. The extracted data included: title, first author, publication year, patient source, country of origin, sample size, total population, age, gender, intervention type, detailed intervention protocol, frequency per day, course of treatment, and outcome measures. Data extraction was independently carried out by two researchers (CQH, WH), followed by cross-checking. Discrepancies were resolved by a third researcher (ZL).

### 2.6 Risk of bias in studies

The Cochrane Collaboration’s tool for evaluating the risk of bias in RCTs was used to determine the bias risk. This tool includes the following seven domains: random sequence generation, allocation concealment, blinding of participants and personnel, blinding of outcome assessment, incomplete outcome data, selective reporting, and other sources of bias. Regarding the generation of the random sequence, some studies failed to provide the complete randomization and methods of random sequence. As a result, the assessment risks for these studies are unclear. Some studies employed pseudo-randomization methods, such as using the parity of admission IDs or grouping based on admission order. These studies were therefore assessed as having a high risk of bias. Regarding allocation concealment, some studies did not employ allocation concealment, leading to a high bias. Other studies employed allocation concealment but did not provide clear descriptions, resulting in an unclear risk of bias. However, some studies utilized opaque envelopes or computer-generated concealment, which were considered to have a low bias. For blinding, some studies implemented single-blind or double-blind procedures, leading to a low bias. Some studies did not employ blinding, resulting in a high risk of bias. In addition, some studies experienced breaches in blinding during implementation, further resulting in a high risk of bias. For the assessment of outcome measures, some studies did not blind assessors, which placed them at a high risk of bias. In contrast, other studies effectively corrected for potential biases in later stages of assessment, resulting in a low risk of bias. Some studies did not provide adequate descriptions of their blinding procedures, leaving the risk of bias unclear. For data integrity, some randomized controlled trials or follow-ups experienced certain data loss. When the data loss is less than 20%, it is generally considered that there is no significant publication bias. The reason for this is that when conducting randomized controlled trials, many studies establish a threshold for follow-up loss of 10%–20% when estimating the number of cases. During selective reporting, some studies set outcomes to be assessed at the onset of the study. If selective reporting of outcomes occurred during the assessment of outcome measures, these studies were considered to have a high risk of bias. Every domain was assessed to have a low, high, or unclear risk of bias. Two researchers (CQH, WH) carried out the risk of bias assessment independently, followed by cross-checking. A third researcher (ZL) was consulted to settle any disagreements.

### 2.7 Synthesis methods

This study aims to compare the advantages of multiple (>2) types of TCM injections in treating UAP. Traditional meta-analysis can only compare the differences between two interventions, and using a subgroup approach to compare multiple interventions can increase the risk of type I errors. Therefore, a network meta-analysis is necessary. Network meta-analysis includes meta-analysis based on frequency and Bayesian theories. In this study, Bayesian network meta-analysis was chosen over the frequentist meta-analysis due to its unique advantages. On the one hand, Bayesian analysis allows for the incorporation of prior knowledge and new data to update the level of confidence for a hypothesis. This provides a more flexible framework for addressing uncertainty in clinical research. In particular, when dealing with studies with small sample sizes or rare diseases, Bayesian methods can utilize results from previous studies as prior distributions, thereby enhancing the capability of statistical inference. Furthermore, Bayesian analysis directly provides a posterior probability distribution, which allows for the direct quantification of parameter estimates and their degree of uncertainty. Therefore, this approach can provide more comprehensive information compared to traditional *P*-values.

The network meta-analysis used a Bayesian random-effects model to compare the efficacy of different interventions. The modeling process was conducted via the Markov Chain Monte Carlo (MCMC) method, with four Markov chains running concurrently. The burn-in process was set to 20,000 iterations, followed by an additional 50,000 iterations to complete the model simulation. The deviance information criterion (DIC) was adopted to assess model fit and global consistency. In cases where the network included closed loops, the node-splitting method was applied to evaluate local consistency. In addition, a league table was created to assess the variations in effect between the different interventions. Treatments were then ordered according to the surface under the cumulative ranking curve (SUCRA). To evaluate study heterogeneity graphically, a funnel plot was used.

Model convergence means that the Markov Chain Monte Carlo (MCMC) algorithm has thoroughly explored the posterior distribution and has reached a steady state. This guarantees the authenticity and representativeness of the sampling results. Convergence is typically assessed by examining Gelman-Rubin diagnostic indicators, observing trace plots, calculating effective sample sizes, and other approaches. If multiple independent chains show similar trends and the R-hat value approaches 1, it indicates successful model convergence. Additionally, to evaluate model performance, its predictive performance and generalizability should be tested. These evaluations can be conducted through cross-validation or by using an independent test set for assessment. Stata v15.0 (Stata Corporation, College Station, TX) and R v4.4.1 (R Development Core Team, Vienna) were employed for the analysis.

## 3 Results

### 3.1 Study selection

**FIGURE 1:**
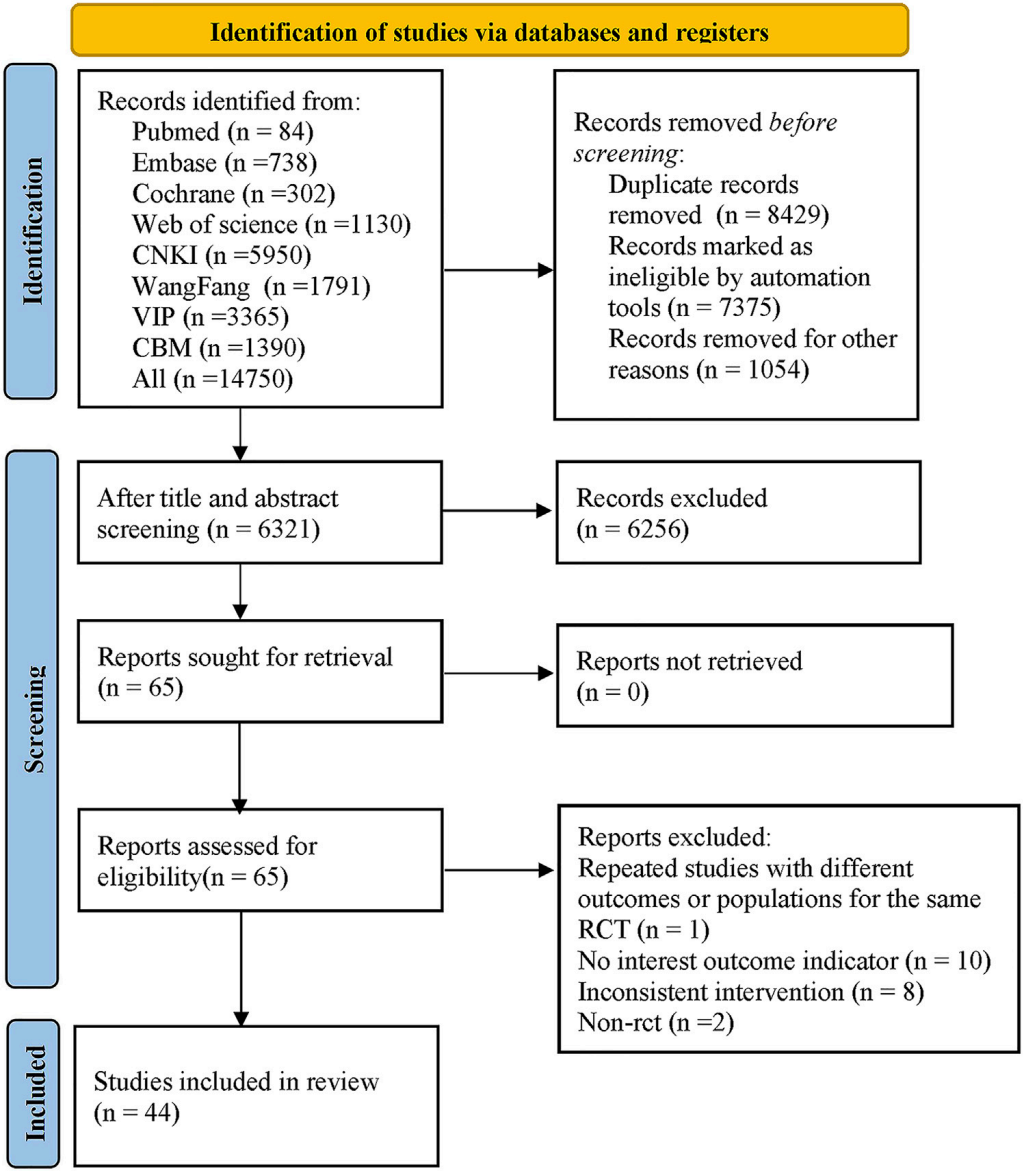
PRISMA flow chart of the literature screening process.

A total of 14,750 articles were retrieved from the databases. After excluding 8,429 duplicates, 65 articles were reviewed by reading their titles and abstracts. The full texts were then downloaded and thoroughly assessed. We excluded 1 study that was a duplicate publication of the same RCT under different outcomes or populations, 10 studies that did not have relevant outcome measures, 8 studies with non-matching interventions, and 2 non-RCT studies. Ultimately, 44 original studies were included in our analysis (Zhang et al., 2022; Miao and Hu, 2013; Gao et al., 2009; Zhang et al., 2019; Ning et al., 2011; Liu et al., 2008; Wang et al., 2002; Chen, 2004; Liu and Zhang, 2022; Wang, 2021; Wang et al., 2012; Yong and Liu, 2010; Gong, 2014; Li and Zhou, 2015; Zhang and Lu, 2016; Wang, 2009; Zhang et al., 2014; Kudereti, 2016; Li and Zhang, 2018; Quan, 2011; Luo, 2014; Li, 2015; Pan et al., 2013; Zhang, 2007; Shi, 2007; Yao and Jin, 2022; Zeng et al., 2015; Yang et al., 2014; Li et al., 2016; Liu et al., 2021; Ren et al., 2018; Qiu et al., 2008; Han and Wen, 2010; Zhou and Liu, 2007; Song and Bannu, 2009; Peng, 2014; Fang, 2006; Chen and Y, 2009; Zheng and Wang, 2006; Zhang, 2006; Pu et al., 2015; Meng, 2004; Zhou, 2008), and the PRISMA flow diagram for searching was displayed in Figure 1.

### 3.2 Study characteristics

The included studies were published between 2002 and 2022 and involved 4,362 patients with UAP. These studies examined 21 different TCM injections. Danhong injection was the most frequently studied, appearing in 12 studies (Miao and Hu, 2013; Gao et al., 2009; Ning et al., 2011; Liu et al., 2008; Gong, 2014; Wang, 2009; Zhang et al., 2014; Li and Zhang, 2018; Pan et al., 2013; Yao and Jin, 2022; Zeng et al., 2015; Zhou, 2008). Other injections included Puerarin (5 studies) (Chen, 2004; Fang, 2006; Chen and Y, 2009; Zheng and Wang, 2006; Meng, 2004), Danshen Chuanxiongqin injection (Li and Zhang, 2018; Yang et al., 2014; Ren et al., 2018), and Xueshuantong injection (Kudereti, 2016; Luo, 2014; Qiu et al., 2008), each appearing in 3 studies. Additional injections studied included Compound Danshen injection (Wang et al., 2002; Song and Bannu, 2009), Danshen injection (Liu et al., 2008; Yong and Liu, 2010), Dengzhanxixin injection (Wang et al., 2012; Yong and Liu, 2010), Ginkgo Damole injection (Quan, 2011; Pu et al., 2015), Shuxuening (Ginkgo Leaf) injection (Wang, 2021; Zhou and Liu, 2007), Gualoupi injection (Zhang, 2007; Song and Bannu, 2009), Ligustrazine hydrochloride injection (Han and Wen, 2010; Peng, 2014), Xingxiong sodium chloride injection (Yao and Jin, 2022; Liu et al., 2021), each appearing in 2 studies, and Guanxinning injection (Zhang, 2006), Hongjingtian injection (Zhang and Lu, 2016), Kudiezi injection (Liu and Zhang, 2022), Safflower yellow pigment injection (Li et al., 2016), Salvianolate injection (Zhai and Jiaol, 2013), Shenmai injection (Li, 2015), Shenxiong glucose injection (Li and Zhou, 2015), Shuxuetong injection (Wang et al., 2002), and Tanshinone injection (Zhang, 2007), each appearing in 1 study. The course of treatment ranged from 2 weeks to 24 weeks (Supplementary Table S2).

### 3.3 Risk of bias in studies

**FIGURE 2:**
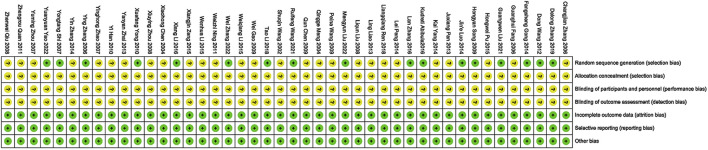
Detailed information on the risk of bias assessment for the studies.

**FIGURE 3:**
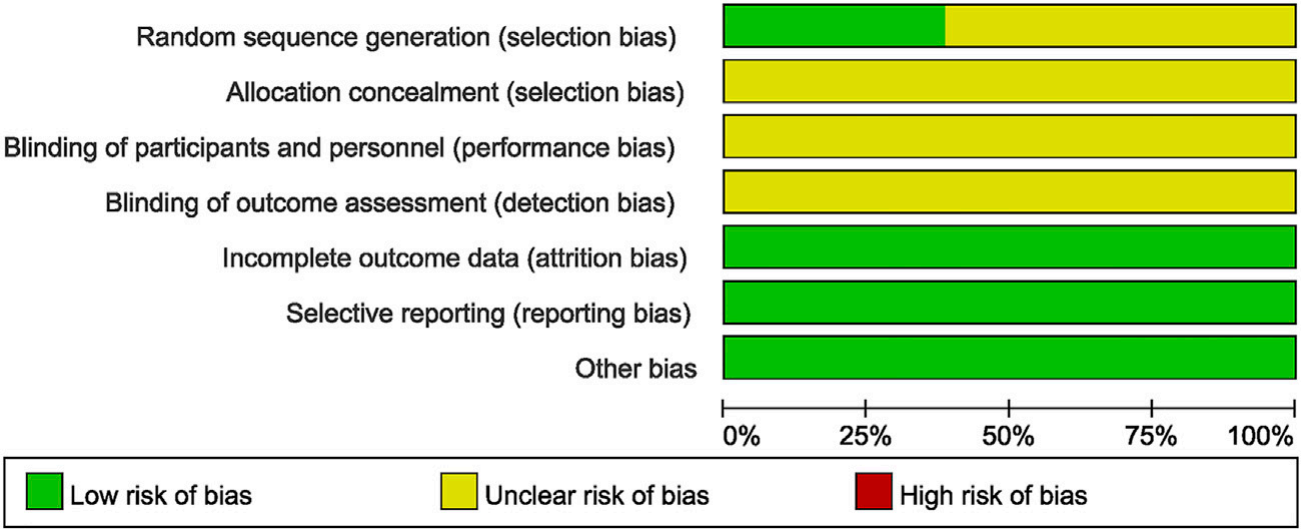
Brief information on the risk assessment of bias in the studies.

All the studies included were RCTs. However, 27 studies described randomization without specifying the exact method used, leading to a classification of low risk of bias. Allocation concealment and blinding were generally not well described, resulting in unclear risk of bias in these domains. There were no significant concerns regarding attrition bias, reporting bias, or other biases, hence these were classified as low risk of bias (Figures 2, 3).

### 3.4 Meta-analysis

#### 3.4.1 Frequency of angina attacks

##### 3.4.1.1 Network diagram of injections

**FIGURE 4:**
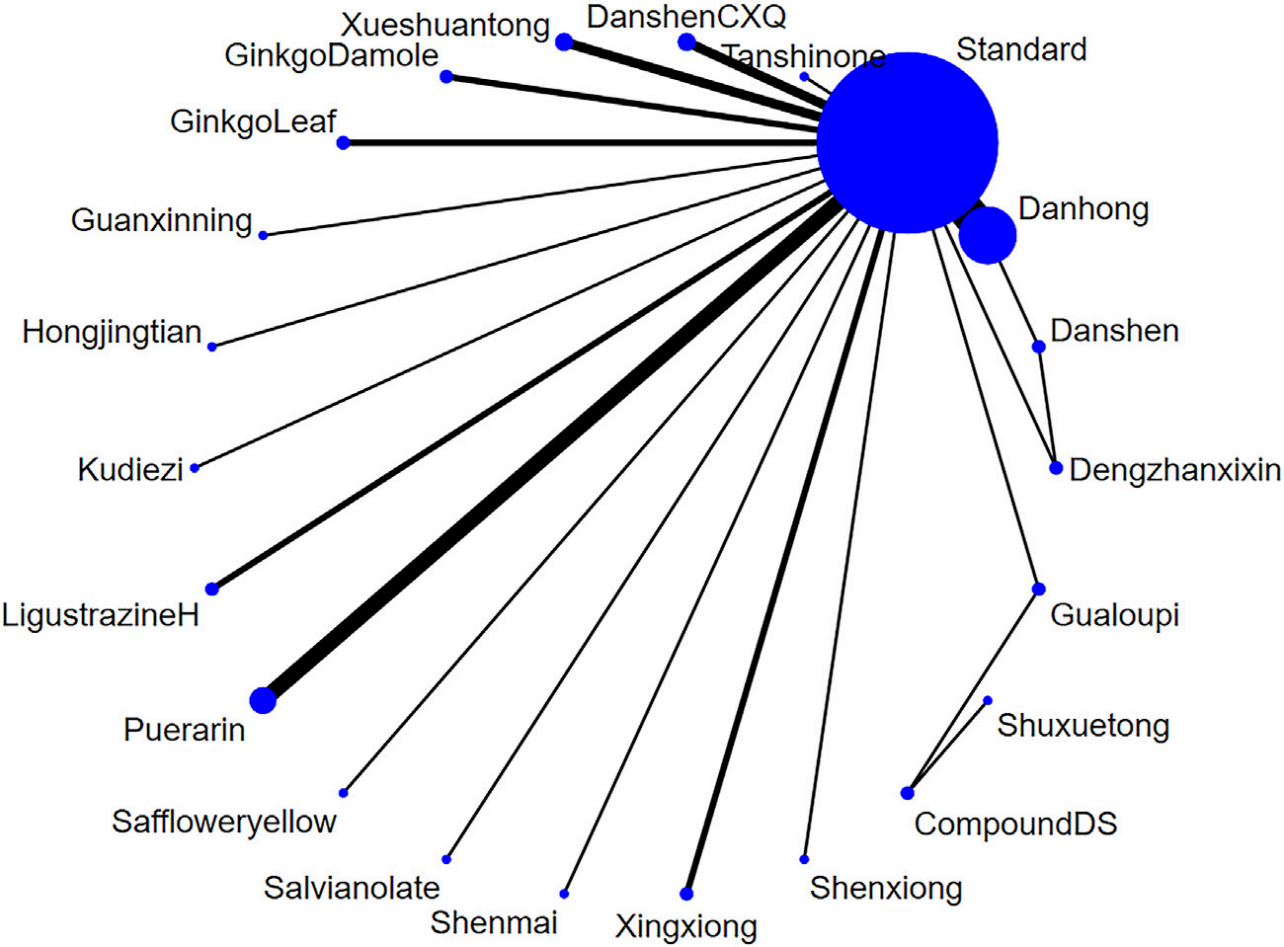
The network relationship of various injections for the frequency of unstable angina pectoris (Note: Each blue circle represents an intervention. A larger diameter indicates that more studies were included for that intervention. A line connecting two blue circles represents a direct comparison between two interventions. A thicker line indicates a higher number of studies comparing the two interventions.).

A total of 44 studies (Zhang et al., 2022; Miao and Hu, 2013; Gao et al., 2009; Zhang et al., 2019; Ning et al., 2011; Liu et al., 2008; Wang et al., 2002; Chen, 2004; Liu and Zhang, 2022; Wang, 2021; Wang et al., 2012; Yong and Liu, 2010; Gong, 2014; Li and Zhou, 2015; Zhang and Lu, 2016; Wang, 2009; Zhang et al., 2014; Kudereti, 2016; Li and Zhang, 2018; Quan, 2011; Luo, 2014; Li, 2015; Pan et al., 2013; Zhang, 2007; Shi, 2007; Yao and Jin, 2022; Zeng et al., 2015; Yang et al., 2014; Li et al., 2016; Liu et al., 2021; Ren et al., 2018; Qiu et al., 2008; Han and Wen, 2010; Zhou and Liu, 2007; Song and Bannu, 2009; Peng, 2014; Fang, 2006; Chen and Y, 2009; Zheng and Wang, 2006; Zhang, 2006; Pu et al., 2015; Meng, 2004; Zhou, 2008) reported on the frequency of angina attacks treated with TCM injections for UAP. Due to differences in the statistical units used, we employed the standardized mean difference (SMD) as the measure for the outcome. The analysis included 21 different TCM injections. The overall heterogeneity of these studies was 4%, and the DIC values for the consistency and inconsistency models were 88.17 and 88.15, respectively, with Danhong injection (Danhong, n = 12), Puerarin injection (Puerarin, n = 5), Danshen Chuanxiongqin injection (DanshenCXQ, n = 3), Xueshuantong injection (Xueshuantong, n = 3), Compound Danshen injection (CompoundDS, n = 2), Danshen injection (Danshen, n = 2), Dengzhanxixin injection (Dengzhanxixin, n = 2), Ginkgo Damole injection (GinkgoDamole, n = 2), Ginkgo Leaf injection (GinkgoLeaf, n = 2), Gualoupi injection (Gualoupi, n = 2), Ligustrazine hydrochloride injection (LigustrazineH, n = 2), Xingxiong sodium chloride injection (Xingxiong, n = 2), Guanxinning injection (Guanxinning, n = 1), Hongjingtian injection (Hongjingtian, n = 1), Kudiezi injection (Kudiezi, n = 1), Safflower yellow pigment injection (Saffloweryellow, n = 1), Salvianolate injection (Salvianolate, n = 1), Shenmai injection (Shenmai, n = 1), Shenxiong glucose injection (Shenxiong, n = 1), Shuxuetong injection (Shuxuetong, n = 1), and Tanshinone injection (Tanshinone, n = 1) included in the network diagram (Figure 4).

##### 3.4.1.2 Results of meta-analysis

**FIGURE 5:**
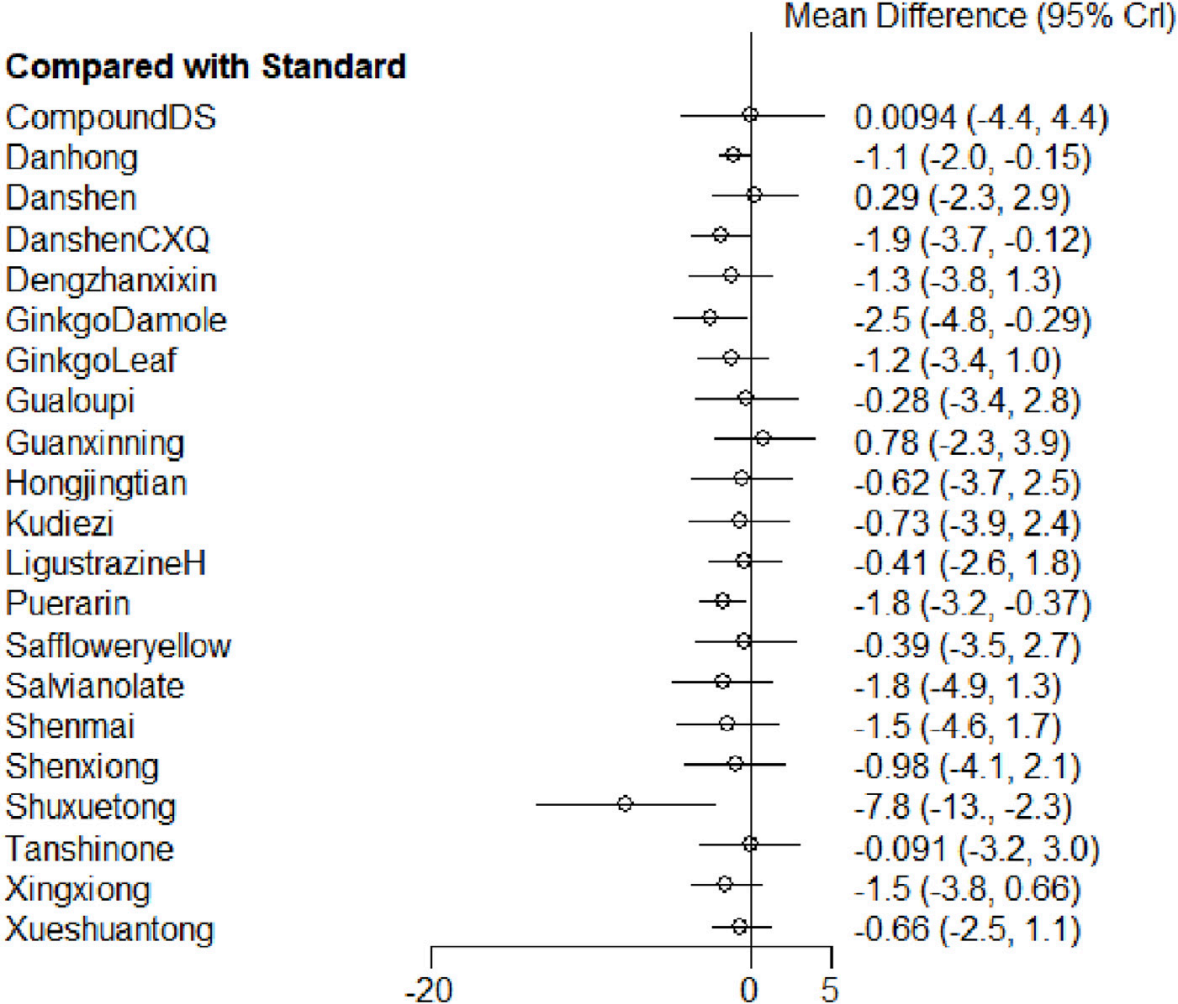
Forest plot of various injections for the frequency of unstable angina pectoris.

The network meta-analysis results indicated that Shuxuetong, Danshen Chuanxiongqin, and Puerarin were the top three interventions in terms of effectiveness for reducing the frequency of angina attacks. Compared with the standard treatment group, Danhong injection (SMD = −1.1, 95% CrI: −2.0, −0.15), Danshen Chuanxiongqin injection (SMD = −1.9, 95% CrI: −3.7, −0.12), Ginkgo Damole injection (SMD = −2.5, 95% CrI: −4.8, −0.29), Puerarin injection (SMD = −1.8, 95% CrI: −3.2, −0.37), and Shuxuetong injection (SMD = −7.8, 95% CrI: −13, −2.3) significantly reduced the frequency of angina attacks (Figure 5; Supplementary Table S3).

#### 3.4.2 Duration of angina attacks

##### 3.4.2.1 Network diagram of injections

**FIGURE 6:**
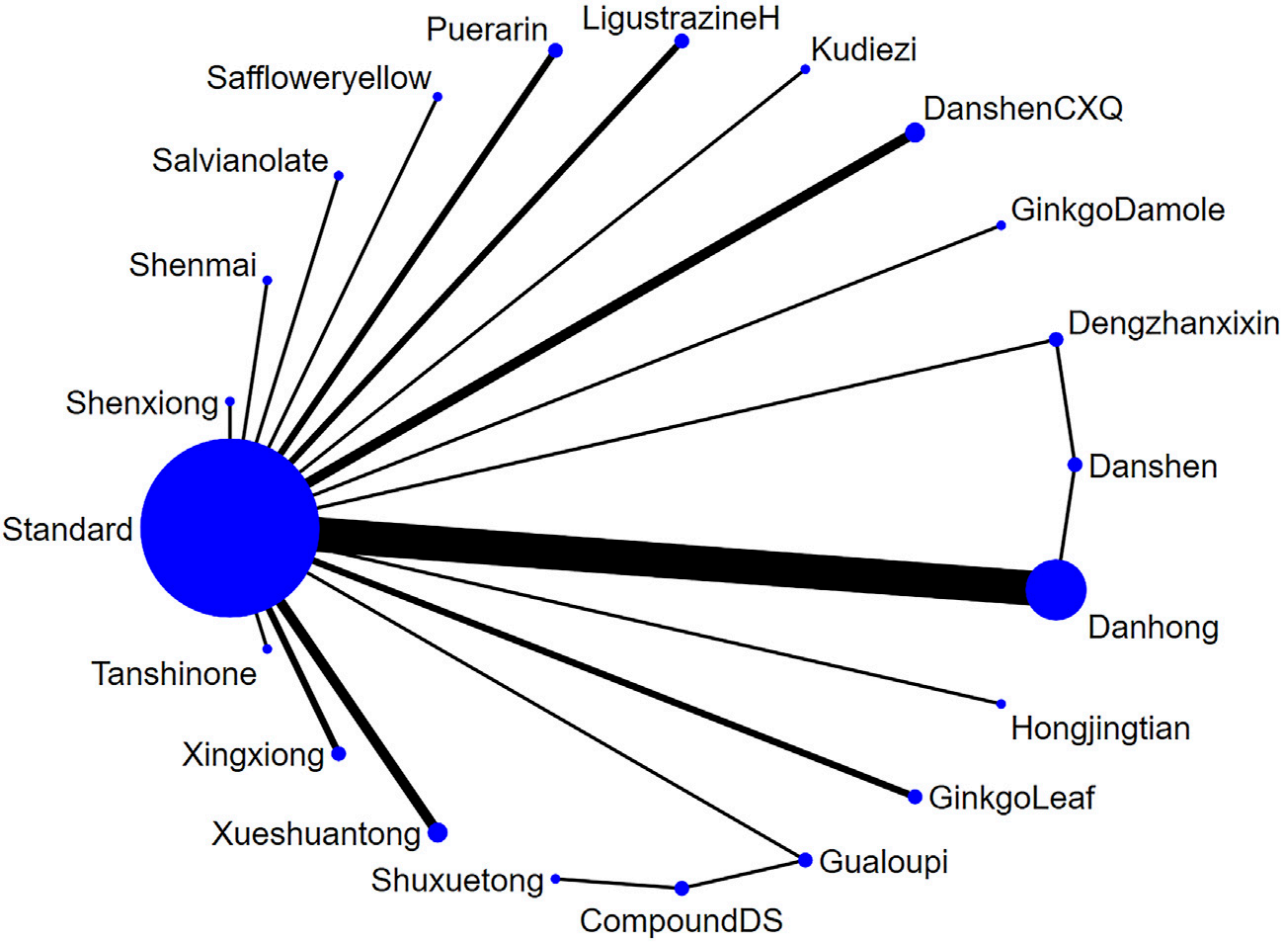
The network relationship of various injections for the duration of unstable angina pectoris. (Note: Each blue circle represents an intervention. A larger diameter indicates that more studies were included for that intervention. A line connecting two blue circles represents a direct comparison between two interventions. A thicker line indicates a higher number of studies comparing the two interventions.)

A total of 38 studies (Zhang et al., 2022; Miao and Hu, 2013; Gao et al., 2009; Zhang et al., 2019; Ning et al., 2011; Liu et al., 2008; Wang et al., 2002; Chen, 2004; Liu and Zhang, 2022; Wang, 2021; Wang et al., 2012; Yong and Liu, 2010; Gong, 2014; Li and Zhou, 2015; Zhang and Lu, 2016; Wang, 2009; Zhang et al., 2014; Kudereti, 2016; Li and Zhang, 2018; Quan, 2011; Luo, 2014; Li, 2015; Pan et al., 2013; Zhang, 2007; Shi, 2007; Yao and Jin, 2022; Zeng et al., 2015; Yang et al., 2014; Li et al., 2016; Liu et al., 2021; Ren et al., 2018; Qiu et al., 2008; Han and Wen, 2010; Zhou and Liu, 2007; Song and Bannu, 2009; Peng, 2014; Fang, 2006; Zhai and Jiaol, 2013) reported on the duration of angina attacks treated with TCM injections for UAP. Due to heterogeneity in the measurement units across studies, SMD was adopted as the measure for the outcome. The analysis included 20 different TCM injections. The overall heterogeneity of these studies was 4%, and the DIC values for the consistency and inconsistency models were 76.21 and 76.14, respectively, with Danhong injection (Danhong, n = 11), Danshen Chuanxiongqin injection (DanshenCXQ, n = 3), Xueshuantong injection (Xueshuantong, n = 3), Compound Danshen injection (CompoundDS, n = 2), Danshen injection (Danshen, n = 2), Puerarin injection (Puerarin, n = 2), Dengzhanxixin injection (Dengzhanxixin, n = 2), Ginkgo Leaf injection (GinkgoLeaf, n = 2), Gualoupi injection (Gualoupi, n = 2), Ligustrazine hydrochloride injection (LigustrazineH, n = 2), Xingxiong sodium chloride injection (Xingxiong, n = 2), Hongjingtian injection (Hongjingtian, n = 1), Kudiezi injection (Kudiezi, n = 1), Ginkgo Damole injection (GinkgoDamole, n = 1), Safflower yellow pigment injection (Saffloweryellow, n = 1), Salvianolate injection (Salvianolate, n = 1), Shenmai injection (Shenmai, n = 1), Shenxiong glucose injection (Shenxiong, n = 1), Shuxuetong injection (Shuxuetong, n = 1), and Tanshinone injection (Tanshinone, n = 1) included in the network diagram (Figure 6).

##### 3.4.2.2 Results of meta-analysis

**FIGURE 7:**
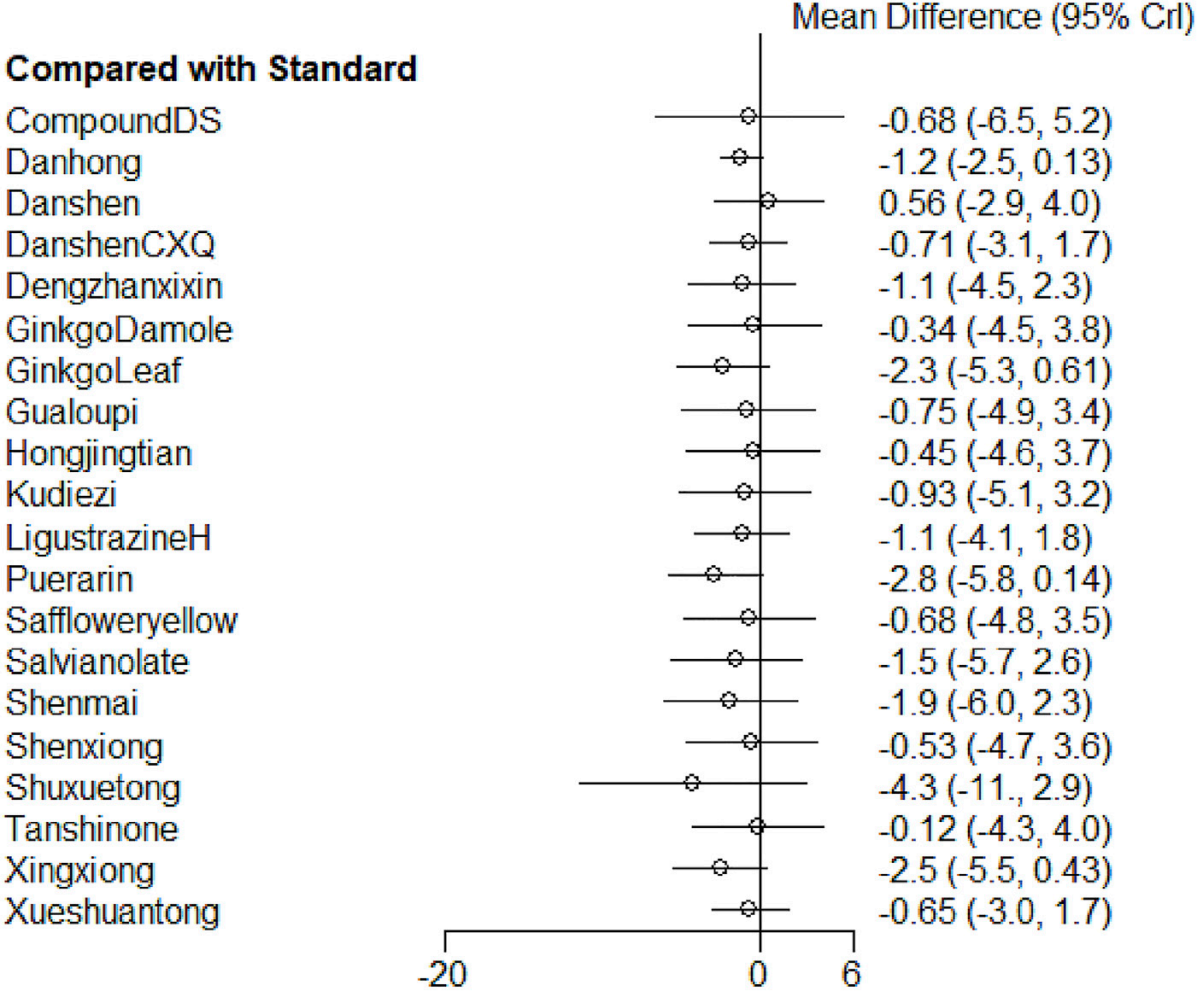
Forest plot of various injections for the duration of unstable angina pectoris.

The network meta-analysis results indicated that Shuxuetong, Puerarin, and Xingxiong were the top three interventions in terms of effectiveness for reducing the duration of angina attacks. However, when comparing the experimental groups receiving TCM injections with the standard treatment group, no notable differences were observed in improving the duration of angina attacks (Figure 7; Supplementary Table S4).

#### 3.4.3 Adverse events

##### 3.4.3.1 Network diagram of injections

**FIGURE 8:**
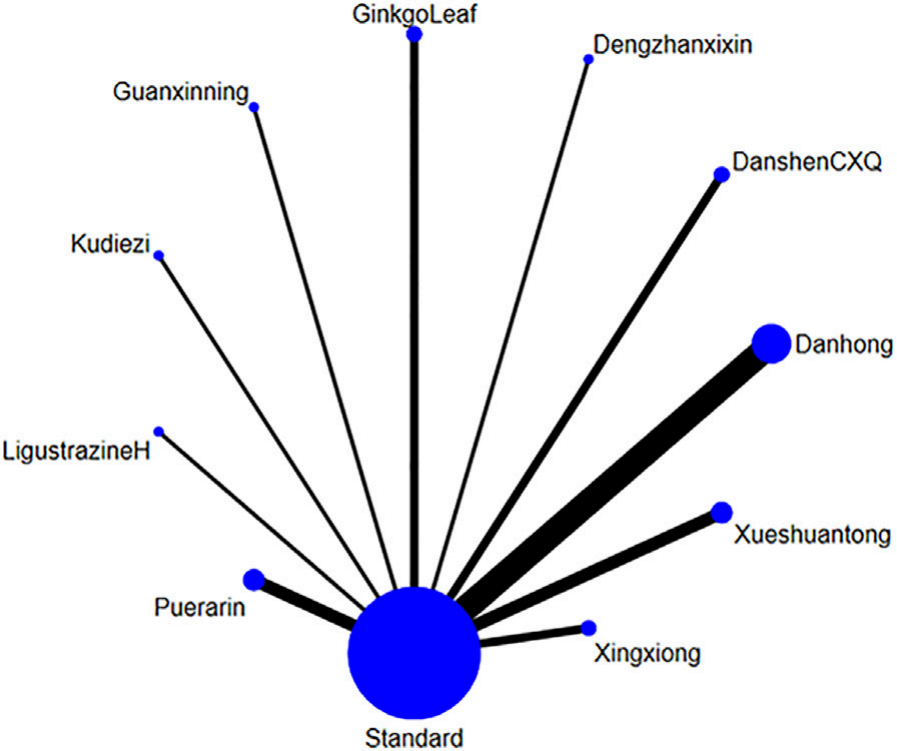
The network relationship of various injections for AEs of unstable angina pectoris. (Note: Each blue circle represents an intervention. A larger diameter indicates that more studies were included for that intervention. A line connecting two blue circles represents a direct comparison between two interventions. A thicker line indicates a higher number of studies comparing the two interventions.)

A total of 22 studies reported adverse events of TCM injections in treating UAP. Adverse events occurred in 14 of 314 subjects treated with Danhong injection, 4 of 126 patients treated with Danshen Chuanxiongqin injection, 3 of 60 patients treated with Dengzhanxin injection, 6 of 145 patients treated with Ginkgo Leaf injection, 2 of 30 patients treated with Guanxinning injection, 6 of 43 patients treated with Kudiezi injection, 1 of 43 patients treated with Ligustrazine hydrochloride injection, 1 of 92 patients treated with Puerarin injection, 7 of 98 patients treated with Xingxiong sodium chloride injection, and 14 of 153 patients treated with Xueshantong injection. Ten injections and standard treatment regimens were included. The overall heterogeneity across these studies was 0%, and the DIC values for the consistency and inconsistency models were 63.53 and 61.84, respectively. Specifically, these include Danhong injection (Danhong, n = 6), Danshen Chuanxiongqin injection (DanshenCXQ, n = 2), Dengzhanxixin injection (Dengzhanxixin, n = 1), Ginkgo Leaf injection (GinkgoLeaf, n = 2), Guanxinning injection (Guanxinning, n = 1), Kudiezi injection (Kudiezi, n = 1), Ligustrazine hydrochloride injection (LigustrazineH, n = 1), Puerarin injection (Puerarin, n = 3), Xingxiong injection (Xingxiong, n = 2), Xueshuantong injection (Xueshuantong, n = 3) and standard treatment regimen (Standard, n = 22) (Figure 8).

##### 3.4.3.2 Results of meta-analysis

**FIGURE 9:**
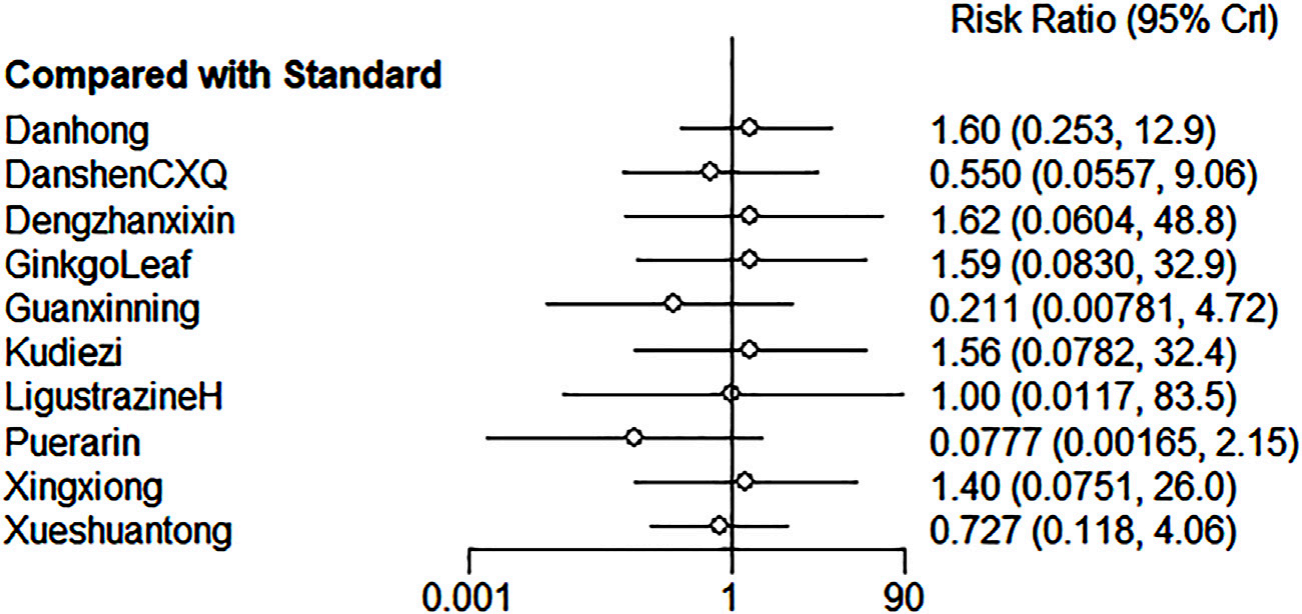
Forest plot of various injections for the AEs of unstable angina pectoris.

The results of the network meta-analysis revealed that there were no significant differences in the risk of adverse events across various TCM injections (Figure 9; Supplementary Table S5).

## 4 Discussion

Currently, there are diverse treatment regimens involving TCM injections for UAP. Our study found that TCM injections demonstrated efficacy in reducing both the frequency and duration of angina attacks. Specifically, Shuxuetong injection, Danshen Chuanxiongqin injection, and Puerarin injection were effective in reducing the frequency of angina attacks, while Shuxuetong injection, Puerarin injection, and Xingxiong sodium chloride injection were effective in reducing the duration of angina attacks.

Pharmacological research indicated that Danhong injection can reduce blood viscosity and increase blood flow velocity, thereby correcting myocardial ischemia by establishing collateral circulation and enhancing coronary blood flow. This mechanism can greatly improve clinical outcomes by reducing the frequency of angina attacks (Qin et al., 2020). A systematic evaluation of Danhong injection for treating ACS demonstrated that, in combination with standard biomedical treatment, Danhong injection more effectively increased the overall efficacy rate, reduced inflammatory cytokines such as high-sensitivity C-reactive protein (hs-CRP) and interleukin-6 (IL-6), lowered plasma viscosity, and reduced levels of plasma endothelin-1 (ET-1) and brain natriuretic peptide (BNP). Additionally, it decreased the production of myeloperoxidase (MPO) and the number of inverted T waves (Wu et al., 2015).

Danshen Chuanxiongqin injection, which contains the active ingredients of Danshensu and ligustrazine hydrochloride, promotes vasodilation to improve myocardial hypoxia and ischemia, inhibit phosphodiesterase activity, and prevents lipid deposition, thereby stabilizing atherosclerotic plaques (Guo, 2018; Bai et al., 2020; Liu and Xu, 2023). A systematic review of Danshen Chuanxiongqin injection showed that its combined use with standard biomedicine was more effective in increasing the total effective rate, especially in improving electrocardiogram (ECG) outcomes, indicating that Danshen Chuanxiongqin injection combined with biomedicine is effective in treating UAP (Zhang X. et al., 2016).

Ginkgo Damole injection, which contains Ginkgo biloba extract and dipyridamole, enhances coronary blood supply and dilation, reduces the rate of apoptosis in damaged cells, and thereby improves myocardial ischemia while maintaining the structural and functional integrity of cells (Yang, 2020; Li, 2019). Additionally, by inhibiting platelets, phosphodiesterase, and thromboxane A2, it also increases endogenous prostacyclin levels, thereby alleviating coronary artery disease symptoms (Fan and Yang, 2021; Feng and Liang, 2019). Xingxiong sodium chloride injection, composed of Ginkgo biloba extract and ligustrazine, has anticoagulant and antithrombotic effects. It can improve microcirculation, act against oxidative free radicals, promote the opening of collateral circulation, and protect ischemic cells. In addition, it can further modulate SDF-1 and CXCR7 expression levels, reduce oxidative stress, alleviate angina symptoms, and improve cardiac function (Zhang et al., 2022; Wu, 2019). Systematic reviews have shown that Ginkgo leaf extract injections (Sun et al., 2015; Liu et al., 2024) are beneficial for patients with angina. These injections have demonstrated efficacy in alleviating symptoms and myocardial ischemia while maintaining a good safety profile.

Puerarin injection (Jiang et al., 2022), primarily composed of an isoflavonoid metabolite derived from Pueraria lobata extract, has a marked effect on dilating coronary arteries. It effectively increases coronary blood flow and reduces vascular resistance (Tan and Li, 2020; Jiang et al., 2020). Puerarin also has antihypertensive properties and can inhibit serotonin (5-HT) release from thrombin-induced platelets, thereby mitigating risk factors that promote the progression of angina (Wang, 2020; He, 2020). Systematic reviews have shown that Puerarin injection (Gao et al., 2015; Shao et al., 2022) is superior to standard biomedicine alone in reducing angina symptoms and improving ECG findings. It also reduces the frequency and duration of angina attacks, nitroglycerin consumption, and plasma endothelin levels. These analyses revealed that the use of Puerarin injection is superior to conventional biomedicine alone in treating UAP.

As another effective TCM preparation, Shuxuetong is composed of *Hirudo* (leech) and *Pheretima* (earthworm), both of which have potent blood-activating and stasis-resolving effects. Modern pharmacological research has shown that hirudin, derived from leech, is the most potent specific thrombin inhibitor currently known (Moher et al., 1998). The extract from earthworms has been found to prolong thrombus formation time in the body and significantly reduce the dry weight and length of thrombi (Guo, 2017).

Safety analysis of the included studies demonstrated that TCM injections exhibited no significant differences in adverse events. Adverse events, such as headaches, dizziness, gastrointestinal reactions, and allergic reactions, were generally well tolerated by patients. In addition, the results of the network meta-analysis demonstrated that the risk of adverse events did not increase with the use of TCM injections compared to standard treatment. There was no significant difference in the incidence of adverse events across different TCM injections. Overall, reasonable use of TCM injections demonstrates favorable safety profiles when properly administered.

Multiple TCM injections are widely applied in the treatment of UAP. This study has demonstrated that the combination of TCM injections with conventional treatment contributes to decreasing the frequency and duration of UAP attacks, with a significant effect on reducing the frequency of angina attacks. The evidence suggests that a reasonable increase in TCM injection use can further improve the symptoms of UAP on the basis of conventional treatment. For patients with more frequent UAP attacks, Danhong injection, Danshen Chuanxiongqin injection, Ginkgo Damole injection, Puerarin injection, and Shuxuetong injection may be considered for adjuvant treatment. Compared to conventional treatment, TCM injections do not increase the incidence of adverse events. Therefore, TCM injections can continue to be used as an adjunct to conventional therapy.

### 4.1 Advantages and limitations of the study

Our research offered a thorough analysis of the effectiveness of different TCM injections for the treatment of UAP. However, several limitations should be noted. First, because TCM is rooted in Chinese culture, the studies included in our analysis were conducted exclusively on Chinese populations. It is hoped that further trials will be carried out with participants from diverse international backgrounds. Second, the studies we included were single-center clinical trials, which may introduce a certain level of bias during the research process. Third, in the studies we included, a substantial number did not report whether allocation concealment or blinding was implemented. As a result, this may pose certain limitations to the interpretation of the results, and further multicenter blinded studies are needed for validation in the future. Fourth, the included studies primarily reported on the frequency and duration of angina attacks, with fewer studies reporting other outcome measures. We hope that future research will explore a broader range of indicators to better assess the efficacy of TCM injections.

## 5 Conclusion

Study results demonstrated that TCM injections showed significant therapeutic effects in the treatment of UAP. When used as an adjunct to conventional treatment, TCM injections reduced the frequency of angina attacks, though they did not significantly decrease the duration of angina episodes. In addition, their use does not lead to a significant increase in the incidence of adverse events.

## Data Availability

The original contributions presented in the study are included in the article/Supplementary Material, further inquiries can be directed to the corresponding author.
