# Supplementary material for: Efficacy and safety of different traditional Chinese medicine injections in the treatment of unstable angina pectoris: a systematic review and Bayesian network meta-analysis
Source: Front Pharmacol. 2025 Mar 12;16:1550759. doi: 10.3389/fphar.2025.1550759 (PMC11937076; doi:10.3389/fphar.2025.1550759)
Supplement: Supplementary file 2 [file Table1.docx]

Table S1: Components of the injections discussed in this study

| **Injections** | **Composition** | **Source** | **Scientific Name** | **Species** | **Family** | **Pharmaceutical producer** |
| --- | --- | --- | --- | --- | --- | --- |
| Ginkgo Leaf injection | *Ginkgo biloba L.* | Leaf of *Ginkgo biloba L.* [Ginkgoaceae] | *Ginkgo biloba L.* | *Ginkgo biloba* | Ginkgoaceae | China Shineway Pharmaceutical Group Limited |
| Xueshuantong injection | Total saponin of panax ginseng | Root of *Panax notoginseng (Burkill) F.H.Chen* [Araliaceae] | *Panax notoginseng (Burkill) F.H.Chen* | *Panax notoginseng* | Araliaceae | Livzon Pharmaceutical Group Limin Pharmaceutical Factory |
| Xingxiong sodium chloride injection | *Ginkgo biloba L.*  tetramethylpyrazine phosphate Each 1 mL contains Ginkgo leaf extract with a total flavonoid glycoside content of 40 μg, and tetramethylpyrazine phosphate (C8H12N2·H3PO4·H2O) 0.4 mg. | Leaf of *Ginkgo biloba L.* [Ginkgoaceae] Root of *Conioselinum anthriscoides ‘Chuanxiong'* [Apiaceae] | *Ginkgo biloba L.  Conioselinum anthriscoides ‘Chuanxiong'* | *Ginkgo biloba Conioselinum anthriscoides* | Ginkgoaceae Apiaceae | Honghe Pharmaceutical Co., Ltd. |
| Kudiezi injection | *Crepidiastrum sonchifolium (Maxim.) Pak & Kawano* | *Whole Crepidiastrum sonchifolium (Maxim.) Pak & Kawano* [Asteraceae] | *Crepidiastrum sonchifolium (Maxim.) Pak & Kawano* | *Crepidiastrum sonchifolium* | Asteraceae | Tonghua Huaxia Pharmaceutical Co., Ltd. |
| Safflower yellow pigment injection | Safflower yellow | Petals of *Carthamus tinctorius L.* [Asteraceae] | *Carthamus tinctorius L.* | *Carthamus tinctorius* | Asteraceae | Zhejiang Yongning Pharmaceutical Co., Ltd. |
| Guanxinning injection | *Salvia miltiorrhiza Bunge*  *Conioselinum anthriscoides ‘Chuanxiong'* | Rhizome of *Salvia miltiorrhiza Bunge* [Lamiaceae] Root of *Conioselinum anthriscoides ‘Chuanxiong'* [Apiaceae] | *Salvia miltiorrhiza Bunge Conioselinum anthriscoides ‘Chuanxiong'* | *Salvia miltiorrhiza Conioselinum anthriscoides* | Lamiaceae Apiaceae | Yabao Pharmaceutical Group Co., Ltd. |
| Gualoupi injection | *Xanthium strumarium L.* | Peel of *Xanthium strumarium L.* [Asteraceae] | *Xanthium strumarium L.* | *Xanthium strumarium* | Asteraceae | SPH No.1 Biochemical & Pharmaceutical Co., Ltd. |
| Compound Danshen injection | *Salvia miltiorrhiza Bunge*  *Dalbergia odorifera T.C.Chen* | Rhizome of *Salvia miltiorrhiza Bunge* [Lamiaceae] Stems of *Dalbergia odorifera T.C.Chen* [Fabaceae] | *Salvia miltiorrhiza Bunge Dalbergia odorifera T.C.Chen* | *Salvia miltiorrhiza Dalbergia odorifera* | Lamiaceae Fabaceae | Shanghai Xinguang Pharmaceutical Factory |
| Puerarin injection | Puerarin | Root of Pueraria montana var.(Willd.) Maesen & S.M. Almeida ex Sanjappa & Predeep | *Pueraria montana var. lobata (Willd.) Maesen & S.M.Almeida ex Sanjappa & Predeep* | *[Pueraria montana](https://powo.science.kew.org/taxon/urn:lsid:ipni.org:names:516708-1)* | Fabaceae | Chengdu TianTaiShan Pharmaceutical Co., Ltd. |
| Dengzhanxixin injection | *Erigeron breviscapus (Vaniot) Hand.-Mazz.* | Whole *Erigeron breviscapus (Vaniot) Hand.-Mazz.* [Asteraceae] | *Erigeron breviscapus (Vaniot) Hand.-Mazz.* | *Erigeron breviscapus* | Asteraceae | Yunnan Biovalley Pharmaceutical Co., Ltd. |
| Danhong injection | *Salvia miltiorrhiza Bunge*  Carthamus tinctorius L. | Rhizome of *Salvia miltiorrhiza Bunge* [Lamiaceae] Petals of *Carthamus tinctorius L.* [Asteraceae] | *Salvia miltiorrhiza Bunge Carthamus tinctorius L.* | *Salvia miltiorrhiza Carthamus tinctorius* | Lamiaceae Asteraceae | Shandong Danhong Pharmaceutical Co., Ltd. |
| Tanshinone injection | tanshinone IIA sodium | Rhizome of *Salvia miltiorrhiza Bunge* [Lamiaceae] | *Salvia miltiorrhiza Bunge* | *Salvia miltiorrhiza* | Lamiaceae | SPH No.1 Biochemical & Pharmaceutical Co., Ltd. |
| Danshen Chuanxiongqin injection | ligustrazine hydrochloride，100mg *Salvia miltiorrhiza Bunge* ，1000mg | Root of *Conioselinum anthriscoides ‘Chuanxiong'* [Apiaceae] Rhizome of *Salvia miltiorrhiza Bunge* [Lamiaceae] | *Conioselinum anthriscoides ‘Chuanxiong'  Salvia miltiorrhiza Bunge* | *Conioselinum anthriscoides Salvia miltiorrhiza* | Apiaceae Lamiaceae | Guizhou Baite Pharmacy Co., Ltd. |
| Ligustrazine hydrochloride injection | ligustrazine hydrochloride | Root of *Conioselinum anthriscoides ‘Chuanxiong'* [Apiaceae] | *Conioselinum anthriscoides ‘Chuanxiong'* | *Conioselinum anthriscoides* | Apiaceae | Suicheng Pharmaceutical Co., Ltd. |
| Shuxuetong injection | *Hirudo nipponica Whitman  Pheretima aspergillum(E.Perrier)* | Whole *Hirudo nipponica Whitman* [Hirudinidae] Whole *Pheretima aspergillum(E.Perrier)*[Megascolecidae] | *Hirudo nipponica Whitman  Pheretima aspergillum(E.Perrier)* | *Hirudo nipponica  Pheretima aspergillum* | Hirudinidae Megascolecidae | Mudanjiang Youbo Pharmaceutical Co., Ltd. |
| Ginkgo Damole injection | Total flavone of Ginkgo dipyridamole Each 5 mL contains 4.5-5.5 mg of total flavone of Ginkgo and 1.8-2.2 mg of dipyridamole. | Leaf of *Ginkgo biloba L.* [Ginkgoaceae] | *Ginkgo biloba L.* | *Ginkgo biloba* | Ginkgoaceae | Guizhou Yibai Pharmaceutical Co., Ltd. |
| Shenxiong glucose injection | ligustrazine hydrochloride Salvianic acid A Each 100 mL contains 100 mg of ligustrazine hydrochloride and 20 mg of salvianic acid A. | Root of *Conioselinum anthriscoides ‘Chuanxiong'* [Apiaceae] Rhizome of *Salvia miltiorrhiza Bunge* [Lamiaceae] | *Conioselinum anthriscoides ‘Chuanxiong'  Salvia miltiorrhiza Bunge* | *Conioselinum anthriscoides Salvia miltiorrhiza* | Apiaceae Lamiaceae | GuiZhou JingFeng Pharmaceutical Co., Ltd. |
| Salvianolate injection | Salvianolate | Rhizome of *Salvia miltiorrhiza Bunge* [Lamiaceae] | *Salvia miltiorrhiza Bunge* | *Salvia miltiorrhiza* | Lamiaceae | Shanghai Green Valley Pharmaceutical Co., Ltd. |
| Hongjingtian injection | *Rhodiola rosea L.* | Root of *Rhodiola rosea L.* [Crassulaceae] | *Rhodiola rosea L.* | *Rhodiola rosea* | Crassulaceae | Tonghua Yusheng Pharmaceutical Co., Ltd. |
| Danshen injection | *Salvia miltiorrhiza Bunge* | Rhizome of *Salvia miltiorrhiza Bunge* [Lamiaceae] | *Salvia miltiorrhiza Bunge* | *Salvia miltiorrhiza* | Lamiaceae | Hubei Minkang Pharmaceutical Co., Ltd. |
| Shenmai injection | *Panax ginseng C.A.Mey.  Ophiopogon japonicus (Thunb.) Ker Gawl.* | Root of *Panax ginseng C.A.Mey.* [Araliaceae] Root of *Ophiopogon japonicus (Thunb.) Ker Gawl.* [Asparagaceae] | *Panax ginseng C.A.Mey. Ophiopogon japonicus (Thunb.) Ker Gawl.* | *Panax ginseng Ophiopogon japonicus* | Araliaceae Asparagaceae | Hebei Shineway Pharmaceutical Group Limited |
