# Supplementary material for: Efficacy and safety of different traditional Chinese medicine injections in the treatment of unstable angina pectoris: a systematic review and Bayesian network meta-analysis
Source: Front Pharmacol. 2025 Mar 12;16:1550759. doi: 10.3389/fphar.2025.1550759 (PMC11937076; doi:10.3389/fphar.2025.1550759)
Supplement: Supplementary file 3 [file Table2.docx]

Table S2: Basic characteristics of studies

| **No.** | **First author** | **Publication year** | **Case No.** | **Total population** | **Age** | **Gender (M/F)** | **Intervention** | **Detailed intervention** | **Treatment duration** |
| --- | --- | --- | --- | --- | --- | --- | --- | --- | --- |
| 1 | Ling Liao | 2013 | T: 30 C: 30 | 60 | T: 58.9±2.8 C: 59.5±2.6 | 49/11 | T: Danghong injection + atorvastatin + routine C: Atorvastatin + routine | Atorvastatin tablet 20 mg orally  Danghong injection 30 mL | 14d |
| 2 | Wei Gao | 2009 | T: 42  C: 40 | 82 | None | None | T: Danghong injection + routine C: Routine | 20mL of Danghong injection added with 10% glucose injection, intravenous infusion of 200mL in total (saline for diabetic patients) | 14d |
| 3 | Delong Zhang | 2019 | T: 59  C: 58 | 117 | T: 59.1 ± 7.3  T: 59.8 ± 7.7 | 85/32 | T: Danshen chuanxiongqin injection + isosorbide mononitrate tablets + routine C: Isosorbide mononitrate tablets + routine | Danshen chuanxiongqin injection 10 ml, 1x/d | 28d |
| 4 | Weizhi Ning | 2011 | T: 25  C: 25 | 50 | T: 62.4  C: 63.1 | 31/19 | T: Danghong injection + routine C: Routine | Danghong injection 20ml | 14d |
| 5 | Liqun Liu | 2008 | T: 148  C: 137 | 285 | None | None | T: Danghong injection + routine C: Dangshen injection + routine | T: Danghong injection 40ml  C: Dangshen injection 20ml | 14d |
| 6 | Shuqin Wang | 2002 | T: 56  C: 53 | 109 | T: 59.2±4.6  C: 58.9±4.8 | 82/27 | T: Shuxuetong injection + routine  C: Compound Danshen + routine | Shuxuetong injection 6ml, Compound Danshen 20ml | 15d |
| 7 | Xiaohong Chen | 2004 | T: 32  C: 27 | 59 | T: 62.5±10.5  C: 62.4±10.2 | 38/21 | T: Puerarin + routine  C: Routine | Puerarin 0.4g | 14d |
| 8 | Mengyun Liu | 2022 | T: 43  C: 43 | 86 | T: 56.33±5.06  C: 56.42±5.28 | 46/40 | T: Kudiezi injection + nicorandil  C: Nicorandil | Kudiezi injection 10-40ml/dose + nicorandil, 14d per course, with a 7d interval | 3m |
| 9 | Wei Zhang | 2022 | T: 46  C: 46 | 92 | T: 50.35±5.04  C: 48.86±5.71 | 49/43 | T: Xingxiong injection + arotinolol hydrochloride tablets + basic treatment C: Arotinolol hydrochloride tablets + basic treatment | Xingxiong injection 250ml/dose, 1x/d + arotinolol hydrochloride tablets 10mg/dose, 2x/d | 14d |
| 10 | Ruifeng Wang | 2021 | T: 65  C: 65 | 130 | T: 62.05±6.59  C: 61.59±6.28 | 69/61 | T: Shuxuening injection + isosorbide mononitrate C: Isosorbide mononitrate | Shuxuening injection 20ml, 1x/d + isosorbide mononitrate | 15d |
| 11 | Qun Chen | 2009 | T: 46  C: 45 | 91 | T: 60.8±7.9  C: 61.1±8.2 | 60/31 | T: Puerarin + routine  C: Routine | Puerarin 500mg | 28d |
| 12 | Ying Zheng | 2006 | T: 30  C: 30 | 60 | T: 58.4  C: 58.2 | 31/29 | T: Puerarin + routine  C: Routine | Puerarin 0.4g + routine | 14d |
| 13 | Changjian Zhang | 2006 | T: 30  C: 30 | 60 | T: 72.43±5.06  C: 68.36±3.65 | 42/18 | T: Guanxinning injection + routine  C: Nitroglycerin injection + routine | Guanxinning injection + routine | 15d |
| 14 | Hongwei Pu | 2015 | T: 42  C: 26 | 68 | None | 41/27 | T: Yinxing damo injection + routine  C: Routine | Yinxing damo injection 20ml + routine | 14d |
| 15 | Dong Wang | 2012 | T: 60  C: 60 | 120 | T: 55.2±8.2  C: 57.8±7.4 | 70/50 | T: Dengzhanxixin injection + routine  C: Routine | Dengzhanxixin injection 40ml + routine | 14d |
| 16 | Xuefang Yong | 2010 | T: 60  C: 60 | 120 | T: 58.43±5.02  C: 57.33±4.69 | 64/56 | T: Dengzhanxixin injection + routine  C: Danshen injection + routine | Dengzhanxixin injection 40ml + routine | 15d |
| 17 | Fangsheng Gong | 2014 | T: 48  C: 48 | 96 | T: 68.22±6.44  C: 69.78±6.5 | 62/34 | T: Danghong injection + routine C: Routine | Danghong injection 30ml + routine | 14d |
| 18 | Qingge Meng | 2004 | T: 30  C: 30 | 60 | T: 60.3±9.2  C: 59.6±10.2 | 31/29 | T: Puerarin injection + routine  C: Routine | Puerarin injection 400mg + routine | 14d |
| 19 | Wenhua Li | 2015 | T: 48  C: 48 | 96 | T: 62.1±5.8  C: 63.2±4.7 | 56/40 | T: Shenxiong glucose injection + routine  C: Routine | Shenxiong glucose injection 100ml + routine | 28d |
| 20 | Yanyan Zhai | 2013 | T: 56  C: 56 | 112 | T: 50.4±8.9  C: 51.3±7.8 | 75/37 | T: Salvianolate + routine  C: Routine | Salvianolate 200mg + routine | 14d |
| 21 | Lan Zhang | 2016 | T: 27  C: 27 | 54 | T: 60±7  C: 60±8 | 39/42 | T: Sofren injection + clopidogrel + routine  C: Clopidogrel + routine | T: Sofren injection 10ml/d + clopidogrel 75mg/d + routine  C: Clopidogrel 75mg/d + routine | 14d |
| 22 | Peina Wang | 2009 | T: 30  C: 30 | 60 | T: 58.5±5.9  C: 58.6±5.7 | 37/23 | T: Danghong injection + routine C: Routine | Danghong injection 30ml + routine | 14d |
| 23 | Xin Zhang | 2014 | T: 80  C: 80 | 160 | T: 59.9±12.9  C: 61.8±12.5 | 78/82 | T: Danghong injection + clopidogrel + routine  C: Clopidogrel + routine | Danghong injection 20ml/d + clopidogrel +routine | 24w |
| 24 | Kudreti Abibula | 2016 | T: 48  C: 48 | 96 | T: 67.4±9.8  C: 66.7±10.5 | 54/42 | T: Xueshuantong injection + nicorandil + routine C: Nicorandil + routine | Xueshuantong injection 490mg + nicorandil + routine | 14d |
| 25 | Tao Li | 2018 | T: 60  C: 60 | 120 | T: 75.8±4.5  C: 75.2±4.9 | 71/49 | T: Danghong injection + routine C: Routine | Danghong injection 40ml + routine | 30d |
| 26 | Zhengrou Quan | 2011 | T: 43  C: 44 | 87 | T: 67.8±7.4  C: 68.4±6.9 | 59/28 | T: Yinxing damo injection + routine  C: Routine | Yinxing damo injection 20ml + routine | 14d |
| 27 | Xiuying Zhou | 2008 | T: 46  C: 48 | 94 | T: 57±12  C: 56±11 | 57/37 | T: Danghong injection + routine C: Routine | Danghong injection 30ml+routine | 14d |
| 28 | Jine Luo | 2014 | T: 75  C: 75 | 150 | T: 60.7±10.1  C: 61.3±10.5 | 79/71 | T: Xueshuantong injection + routine  C: Routine | Xueshuantong injection 500mg + routine | 14d |
| 29 | Weiqiang Li | 2015 | T: 30  C: 30 | 60 | T: 63.8±12.8  C: 64.2±12.5 | 38/22 | T: Shenmai injection + routine  C: Routine | Shenmai injection 40ml + routine | 15d |
| 30 | Juxiang Pan | 2013 | T: 63  C: 63 | 126 | ALL: 53.63±6.32 | 72/54 | T: Danghong injection + atorvastatin + routine  C: Atorvastatin + routine | Danghong injection 30ml + atorvastatin 20mg, 1x/d +routine | 28d |
| 31 | Yinghong Zhao | 2007 | T: 39  C: 39 | 78 | ALL:  62.8±6.7 | 54/24 | T: Tanshinone injection  C: Isosorbide mononitrate injection | Tanshinone injection 40mg | 14d |
| 32 | Yongtang Shi | 2007 | T: 56  C: 54 | 110 | T: 58.6±6  C: 59.2±7 | 61/49 | T: Xintong injection + low-molecular-weight heparin calcium +routine  C: Low-molecular-weight heparin calcium +routine | Xintong injection 10ml, 1x/d + low-molecular-weight heparin calcium 5000IU, every 12h for 7d + routine | 14d |
| 33 | Yuanyuan Yao | 2022 | T: 56  C: 56 | 112 | T: 58.43±7.56  C: 57.69±7.41 | 65/47 | T: Danghong injection + routine C: Routine | Danghong injection 20ml + routine | 28d |
| 34 | Xiangjin Zeng | 2015 | T: 75  C: 75 | 150 | None | None | T: Danghong injection + routine C: Routine | Danghong injection 20ml + routine  C: Routine | 45d |
| 35 | Kai Yang | 2014 | T: 72  C: 65 | 137 | T: 55±18.9  C: 51±19.3 | 80/57 | T: Danshen chuanxiongqin injection + routine C: Routine | Danshen chuanxiongqin injection 10ml + routine | 15d |
| 36 | Xiang Li | 2016 | T: 43  C: 43 | 86 | T: 60.13±3.29  C: 60.21±3.08 | 53/33 | T: Safflower yellow + atorvastatin calcium  C: atorvastatin calcium | Safflower yellow 100mg + atorvastatin calcium 20mg | 14d |
| 37 | Guangwen Liu | 2021 | T: 52  C: 52 | 104 | T: 51.84±1.57  C: 51.43±1.25 | 56/48 | T: Xingxiong sodium chloride injection + ticagrelor tablets  C: Ticagrelor tablets | Intravenous infusion of xingxiong sodium chloride injection, 250 mL/dose, 1x | 14d |
| 38 | Liangqiang Ren | 2018 | T: 54  C: 54 | 108 | T: 54.97±11.29  C: 53.92±12.78 | 61/47 | T: Danshen chuanxiongqin injection + ticagrelor tablets +routine  C: Ticagrelor tablets +routine | Dripping danshen chuanxiongqin injection, 10 mL | 14d |
| 39 | Zhenwei Qiu | 2008 | T: 30  C: 30 | 60 | T: 71.25  C: 71.44 | 39/21 | T: Xueshuantong injection +routine  C: Isosorbide dinitrate +routine | Xueshuantong injection 500mg + routine | 14d |
| 40 | Yi Han | 2010 | T: 32  C: 28 | 60 | ALL:  65 | 38/22 | T: Ligustrazine hydrochloride injection + routine  C: Isosorbide mononitrate disperible + routine | Ligustrazine hydrochloride injection 160mg + routine | 14d |
| 41 | Yuming Zhou | 2007 | T: 80  C: 70 | 150 | T: 60.4±12.8  C: 61.7±11.2 | 75/75 | T: Ginkgo biloba injection +routine  C: Routine | Ginkgo biloba injection 20ml + routine | 15d |
| 42 | Hongyan Song | 2009 | T: 54  C: 48 | 102 | T: 61.3±4.41  C: 59.8±9.63 | 53/49 | T: Pericarpium trichosanthis injection + routine  C: Compound danshen injection + routine | Pericarpium trichosanthis 12ml + routine | 14d |
| 43 | Lei Peng | 2014 | T: 43  C: 42 | 85 | ALL: 61.2±5.94 | 54/31 | T: Ligustrazine hydrochloride injection + routine  C: Routine | Ligustrazine hydrochloride injection 80mg + routine | 14d |
| 44 | Guanghui Fang | 2006 | T: 30  C: 30 | 60 | T: 71.36  C: 71.46 | 41/19 | T: Puerarin injection +routine  C: Isosorbide mononitrate disperible + routine | Puerarin injection 400mg+ routine | 14d |
