# Supplementary material for: Efficacy and safety of different traditional Chinese medicine injections in the treatment of unstable angina pectoris: a systematic review and Bayesian network meta-analysis
Source: Front Pharmacol. 2025 Mar 12;16:1550759. doi: 10.3389/fphar.2025.1550759 (PMC11937076; doi:10.3389/fphar.2025.1550759)
Supplement: Supplementary file 5 [file Table5.docx]

Table S5: League table of various injections for reducing the AEs in individuals with UAP

|  | Danhong | DanshenCXQ | Dengzhanxixin | GinkgoLeaf | Guanxinning | Kudiezi | LigustrazineH | Puerarin | Standard | Xingxiong | Xueshuantong |
| --- | --- | --- | --- | --- | --- | --- | --- | --- | --- | --- | --- |
| Danhong | Danhong | 0.341 (0.016, 9.47) | 1.004 (0.019, 46.435) | 0.992 (0.025, 32.246) | 0.131 (0.003, 4.617) | 0.976 (0.024, 31.333) | 0.616 (0.004, 73.428) | 0.047 (0.001, 2.055) | 0.625 (0.078, 3.945) | 0.88 (0.023, 25.679) | 0.454 (0.027, 5.451) |
| DanshenCXQ | 2.935 (0.106, 61.316) | DanshenCXQ | 2.95 (0.037, 165.281) | 2.915 (0.047, 115.926) | 0.38 (0.005, 16.345) | 2.869 (0.044, 113.511) | 1.767 (0.009, 250.531) | 0.137 (0.001, 7.215) | 1.818 (0.11, 17.946) | 2.591 (0.041, 92.097) | 1.328 (0.044, 21.716) |
| Dengzhanxixin | 0.996 (0.022, 51.932) | 0.339 (0.006, 27.359) | Dengzhanxixin | 0.983 (0.011, 84.591) | 0.127 (0.001, 12.087) | 0.948 (0.011, 84.027) | 0.611 (0.002, 152.294) | 0.046 (0, 5.299) | 0.617 (0.02, 16.556) | 0.86 (0.01, 69.941) | 0.447 (0.009, 18.162) |
| GinkgoLeaf | 1.008 (0.031, 40.03) | 0.343 (0.009, 21.489) | 1.017 (0.012, 93.041) | GinkgoLeaf | 0.13 (0.002, 9.699) | 0.981 (0.014, 69.019) | 0.633 (0.003, 128.155) | 0.047 (0, 4.103) | 0.63 (0.03, 12.045) | 0.888 (0.013, 55.213) | 0.457 (0.013, 13.936) |
| Guanxinning | 7.648 (0.217, 396.478) | 2.628 (0.061, 212.647) | 7.853 (0.083, 863.815) | 7.668 (0.103, 661.349) | Guanxinning | 7.521 (0.1, 645.757) | 4.841 (0.021, 1161.27) | 0.361 (0.003, 40.089) | 4.729 (0.212, 128.016) | 6.716 (0.092, 533.674) | 3.437 (0.093, 140.031) |
| Kudiezi | 1.025 (0.032, 41.52) | 0.349 (0.009, 22.703) | 1.054 (0.012, 94.293) | 1.019 (0.014, 70.798) | 0.133 (0.002, 10.023) | Kudiezi | 0.635 (0.003, 130.54) | 0.049 (0, 4.314) | 0.643 (0.031, 12.788) | 0.897 (0.013, 57.491) | 0.467 (0.013, 14.406) |
| LigustrazineH | 1.624 (0.014, 223.646) | 0.566 (0.004, 109.597) | 1.636 (0.007, 418.666) | 1.58 (0.008, 340.302) | 0.207 (0.001, 47.596) | 1.576 (0.008, 334.264) | LigustrazineH | 0.074 (0, 20.495) | 0.998 (0.012, 85.598) | 1.395 (0.007, 273.3) | 0.719 (0.006, 84.132) |
| Puerarin | 21.109 (0.487, 1715.088) | 7.318 (0.139, 876.658) | 21.688 (0.189, 3473.982) | 21.215 (0.244, 2575.942) | 2.766 (0.025, 372.855) | 20.61 (0.232, 2664.542) | 13.475 (0.049, 4388.109) | Puerarin | 12.877 (0.466, 606.944) | 18.433 (0.21, 2153.702) | 9.421 (0.205, 608.801) |
| Standard | 1.601 (0.253, 12.881) | 0.55 (0.056, 9.058) | 1.621 (0.06, 48.844) | 1.586 (0.083, 32.919) | 0.211 (0.008, 4.719) | 1.555 (0.078, 32.371) | 1.002 (0.012, 83.502) | 0.078 (0.002, 2.145) | Standard | 1.401 (0.075, 26.015) | 0.727 (0.118, 4.064) |
| Xingxiong | 1.137 (0.039, 44.35) | 0.386 (0.011, 24.473) | 1.163 (0.014, 101.27) | 1.126 (0.018, 77.858) | 0.149 (0.002, 10.833) | 1.115 (0.017, 74.387) | 0.717 (0.004, 141.449) | 0.054 (0, 4.771) | 0.714 (0.038, 13.311) | Xingxiong | 0.514 (0.016, 15.154) |
| Xueshuantong | 2.203 (0.183, 36.447) | 0.753 (0.046, 22.644) | 2.237 (0.055, 108.406) | 2.187 (0.072, 76.323) | 0.291 (0.007, 10.758) | 2.142 (0.069, 74.818) | 1.391 (0.012, 166.294) | 0.106 (0.002, 4.882) | 1.376 (0.246, 8.475) | 1.945 (0.066, 60.794) | Xueshuantong |

Note: Risk Radio was used to calculate the effect size of AEs between groups. When 95% CrI contains 1, the difference is not significant.
