# Supplementary material for: Efficacy and safety of different traditional Chinese medicine injections in the treatment of unstable angina pectoris: a systematic review and Bayesian network meta-analysis
Source: Front Pharmacol. 2025 Mar 12;16:1550759. doi: 10.3389/fphar.2025.1550759 (PMC11937076; doi:10.3389/fphar.2025.1550759)
Supplement: Supplementary file 6 [file Table3.docx]

Table S3: League table of various injections for treating UAP with angina attack frequency as the outcome

|  | **CompoundDS** | **Danhong** | **Danshen** | **DanshenCXQ** | **Dengzhanxixin** | **GinkgoDamole** | **GinkgoLeaf** | **Gualoupi** | **Guanxinning** | **Hongjingtian** | **Kudiezi** | **LigustrazineH** | **Puerarin** | **Saffloweryellow** | **Salvianolate** | **Shenmai** | **Shenxiong** | **Shuxuetong** | **Standard** | **Tanshinone** | **Xingxiong** | **Xueshuantong** |  |
| --- | --- | --- | --- | --- | --- | --- | --- | --- | --- | --- | --- | --- | --- | --- | --- | --- | --- | --- | --- | --- | --- | --- | --- |
| CompoundDS | CompoundDS | -1.09 (-5.62, 3.4) | 0.27 (-4.87, 5.4) | -1.93 (-6.71, 2.84) | -1.29 (-6.41, 3.8) | -2.53 (-7.5, 2.4) | -1.17 (-6.12, 3.74) | -0.3 (-3.4, 2.82) | 0.77 (-4.65, 6.15) | -0.62 (-6.06, 4.77) | -0.74 (-6.13, 4.63) | -0.43 (-5.34, 4.51) | -1.78 (-6.42, 2.84) | -0.4 (-5.8, 5) | -1.81 (-7.22, 3.56) | -1.48 (-6.92, 3.9) | -0.99 (-6.41, 4.38) | -7.83 (-11.13, -4.54) | -0.01 (-4.42, 4.39) | -0.1 (-5.51, 5.27) | -1.56 (-6.5, 3.37) | -0.68 (-5.44, 4.1) |  |
| Danhong | 1.09 (-3.4, 5.62) | Danhong | 1.36 (-1.19, 3.93) | -0.83 (-2.86, 1.19) | -0.2 (-2.81, 2.41) | -1.44 (-3.87, 0.97) | -0.08 (-2.47, 2.31) | 0.79 (-2.46, 4.05) | 1.86 (-1.4, 5.12) | 0.47 (-2.79, 3.73) | 0.35 (-2.9, 3.61) | 0.66 (-1.73, 3.08) | -0.69 (-2.4, 1) | 0.69 (-2.55, 3.95) | -0.72 (-3.99, 2.54) | -0.39 (-3.66, 2.88) | 0.09 (-3.16, 3.35) | -6.74 (-12.31, -1.15) | 1.08 (0.15, 2.01) | 0.99 (-2.29, 4.22) | -0.47 (-2.88, 1.93) | 0.42 (-1.61, 2.44) |  |
| Danshen | -0.27 (-5.4, 4.87) | -1.36 (-3.93, 1.19) | Danshen | -2.2 (-5.38, 0.96) | -1.57 (-4.12, 1.01) | -2.8 (-6.25, 0.64) | -1.45 (-4.87, 1.96) | -0.56 (-4.65, 3.52) | 0.5 (-3.6, 4.6) | -0.9 (-4.96, 3.19) | -1.02 (-5.09, 3.07) | -0.7 (-4.14, 2.74) | -2.05 (-5.05, 0.9) | -0.67 (-4.74, 3.4) | -2.08 (-6.16, 1.99) | -1.75 (-5.84, 2.34) | -1.27 (-5.35, 2.82) | -8.1 (-14.19, -2.01) | -0.29 (-2.91, 2.34) | -0.38 (-4.46, 3.69) | -1.83 (-5.25, 1.59) | -0.94 (-4.13, 2.22) |  |
| DanshenCXQ | 1.93 (-2.84, 6.71) | 0.83 (-1.19, 2.86) | 2.2 (-0.96, 5.38) | DanshenCXQ | 0.63 (-2.49, 3.77) | -0.61 (-3.48, 2.26) | 0.75 (-2.08, 3.57) | 1.62 (-1.96, 5.25) | 2.69 (-0.9, 6.31) | 1.3 (-2.3, 4.92) | 1.18 (-2.42, 4.77) | 1.5 (-1.35, 4.34) | 0.14 (-2.15, 2.41) | 1.52 (-2.08, 5.12) | 0.11 (-3.48, 3.71) | 0.45 (-3.16, 4.06) | 0.92 (-2.66, 4.52) | -5.9 (-11.69, -0.13) | 1.91 (0.12, 3.71) | 1.82 (-1.77, 5.39) | 0.37 (-2.48, 3.21) | 1.25 (-1.3, 3.8) |  |
| Dengzhanxixin | 1.29 (-3.8, 6.41) | 0.2 (-2.41, 2.81) | 1.57 (-1.01, 4.12) | -0.63 (-3.77, 2.49) | Dengzhanxixin | -1.24 (-4.65, 2.16) | 0.11 (-3.26, 3.49) | 1 (-3.05, 5.06) | 2.06 (-1.99, 6.12) | 0.67 (-3.37, 4.7) | 0.55 (-3.47, 4.57) | 0.86 (-2.52, 4.25) | -0.49 (-3.43, 2.42) | 0.89 (-3.14, 4.94) | -0.52 (-4.56, 3.51) | -0.19 (-4.24, 3.86) | 0.29 (-3.74, 4.34) | -6.54 (-12.63, -0.48) | 1.28 (-1.28, 3.84) | 1.18 (-2.88, 5.23) | -0.27 (-3.64, 3.12) | 0.62 (-2.52, 3.75) |  |
| GinkgoDamole | 2.53 (-2.4, 7.5) | 1.44 (-0.97, 3.87) | 2.8 (-0.64, 6.25) | 0.61 (-2.26, 3.48) | 1.24 (-2.16, 4.65) | GinkgoDamole | 1.36 (-1.76, 4.51) | 2.23 (-1.6, 6.09) | 3.3 (-0.53, 7.16) | 1.9 (-1.92, 5.76) | 1.79 (-2.03, 5.62) | 2.1 (-1.02, 5.26) | 0.74 (-1.89, 3.4) | 2.12 (-1.69, 6) | 0.72 (-3.11, 4.56) | 1.05 (-2.79, 4.9) | 1.53 (-2.29, 5.37) | -5.31 (-11.25, 0.68) | 2.51 (0.29, 4.77) | 2.42 (-1.41, 6.27) | 0.97 (-2.17, 4.13) | 1.86 (-1.01, 4.74) |  |
| GinkgoLeaf | 1.17 (-3.74, 6.12) | 0.08 (-2.31, 2.47) | 1.45 (-1.96, 4.87) | -0.75 (-3.57, 2.08) | -0.11 (-3.49, 3.26) | -1.36 (-4.51, 1.76) | GinkgoLeaf | 0.88 (-2.92, 4.69) | 1.94 (-1.86, 5.76) | 0.55 (-3.25, 4.38) | 0.43 (-3.36, 4.24) | 0.75 (-2.37, 3.88) | -0.61 (-3.23, 1.99) | 0.77 (-3.03, 4.59) | -0.63 (-4.45, 3.17) | -0.31 (-4.13, 3.51) | 0.18 (-3.63, 3.99) | -6.65 (-12.6, -0.74) | 1.16 (-1.03, 3.37) | 1.07 (-2.75, 4.87) | -0.39 (-3.5, 2.75) | 0.5 (-2.33, 3.35) |  |
| Gualoupi | 0.3 (-2.82, 3.4) | -0.79 (-4.05, 2.46) | 0.56 (-3.52, 4.65) | -1.62 (-5.25, 1.96) | -1 (-5.06, 3.05) | -2.23 (-6.09, 1.6) | -0.88 (-4.69, 2.92) | Gualoupi | 1.06 (-3.35, 5.45) | -0.33 (-4.74, 4.09) | -0.44 (-4.86, 3.94) | -0.13 (-3.95, 3.68) | -1.49 (-4.92, 1.92) | -0.11 (-4.5, 4.31) | -1.52 (-5.93, 2.88) | -1.18 (-5.63, 3.23) | -0.7 (-5.11, 3.71) | -7.53 (-12.08, -3.04) | 0.28 (-2.82, 3.4) | 0.19 (-4.22, 4.57) | -1.26 (-5.09, 2.57) | -0.38 (-3.98, 3.21) |  |
| Guanxinning | -0.77 (-6.15, 4.65) | -1.86 (-5.12, 1.4) | -0.5 (-4.6, 3.6) | -2.69 (-6.31, 0.9) | -2.06 (-6.12, 1.99) | -3.3 (-7.16, 0.53) | -1.94 (-5.76, 1.86) | -1.06 (-5.45, 3.35) | Guanxinning | -1.4 (-5.8, 3.04) | -1.51 (-5.92, 2.88) | -1.2 (-5.03, 2.63) | -2.55 (-6, 0.86) | -1.18 (-5.59, 3.24) | -2.57 (-7, 1.82) | -2.25 (-6.69, 2.16) | -1.77 (-6.18, 2.64) | -8.6 (-14.91, -2.28) | -0.78 (-3.9, 2.33) | -0.88 (-5.28, 3.53) | -2.32 (-6.16, 1.49) | -1.44 (-5.05, 2.15) |  |
| Hongjingtian | 0.62 (-4.77, 6.06) | -0.47 (-3.73, 2.79) | 0.9 (-3.19, 4.96) | -1.3 (-4.92, 2.3) | -0.67 (-4.7, 3.37) | -1.9 (-5.76, 1.92) | -0.55 (-4.38, 3.25) | 0.33 (-4.09, 4.74) | 1.4 (-3.04, 5.8) | Hongjingtian | -0.12 (-4.54, 4.27) | 0.2 (-3.64, 4.02) | -1.16 (-4.6, 2.25) | 0.22 (-4.18, 4.62) | -1.19 (-5.6, 3.21) | -0.86 (-5.3, 3.55) | -0.36 (-4.8, 4.03) | -7.2 (-13.56, -0.88) | 0.62 (-2.53, 3.74) | 0.52 (-3.91, 4.9) | -0.94 (-4.75, 2.88) | -0.05 (-3.66, 3.54) |  |
| Kudiezi | 0.74 (-4.63, 6.13) | -0.35 (-3.61, 2.9) | 1.02 (-3.07, 5.09) | -1.18 (-4.77, 2.42) | -0.55 (-4.57, 3.47) | -1.79 (-5.62, 2.03) | -0.43 (-4.24, 3.36) | 0.44 (-3.94, 4.86) | 1.51 (-2.88, 5.92) | 0.12 (-4.27, 4.54) | Kudiezi | 0.31 (-3.51, 4.15) | -1.04 (-4.46, 2.38) | 0.34 (-4.07, 4.75) | -1.07 (-5.48, 3.32) | -0.75 (-5.16, 3.68) | -0.26 (-4.64, 4.14) | -7.08 (-13.41, -0.77) | 0.73 (-2.38, 3.85) | 0.63 (-3.77, 5.03) | -0.82 (-4.64, 3) | 0.07 (-3.53, 3.67) |  |
| LigustrazineH | 0.43 (-4.51, 5.34) | -0.66 (-3.08, 1.73) | 0.7 (-2.74, 4.14) | -1.5 (-4.34, 1.35) | -0.86 (-4.25, 2.52) | -2.1 (-5.26, 1.02) | -0.75 (-3.88, 2.37) | 0.13 (-3.68, 3.95) | 1.2 (-2.63, 5.03) | -0.2 (-4.02, 3.64) | -0.31 (-4.15, 3.51) | LigustrazineH | -1.36 (-4.01, 1.27) | 0.03 (-3.82, 3.85) | -1.38 (-5.2, 2.43) | -1.06 (-4.9, 2.79) | -0.57 (-4.38, 3.26) | -7.4 (-13.36, -1.49) | 0.41 (-1.81, 2.62) | 0.32 (-3.53, 4.13) | -1.13 (-4.28, 2) | -0.24 (-3.11, 2.6) |  |
| Puerarin | 1.78 (-2.84, 6.42) | 0.69 (-1, 2.4) | 2.05 (-0.9, 5.05) | -0.14 (-2.41, 2.15) | 0.49 (-2.42, 3.43) | -0.74 (-3.4, 1.89) | 0.61 (-1.99, 3.23) | 1.49 (-1.92, 4.92) | 2.55 (-0.86, 6) | 1.16 (-2.25, 4.6) | 1.04 (-2.38, 4.46) | 1.36 (-1.27, 4.01) | Puerarin | 1.38 (-2.04, 4.83) | -0.03 (-3.44, 3.4) | 0.31 (-3.14, 3.74) | 0.78 (-2.63, 4.21) | -6.04 (-11.72, -0.38) | 1.77 (0.37, 3.2) | 1.68 (-1.75, 5.09) | 0.22 (-2.4, 2.85) | 1.11 (-1.17, 3.4) |  |
| Saffloweryellow | 0.4 (-5, 5.8) | -0.69 (-3.95, 2.55) | 0.67 (-3.4, 4.74) | -1.52 (-5.12, 2.08) | -0.89 (-4.94, 3.14) | -2.12 (-6, 1.69) | -0.77 (-4.59, 3.03) | 0.11 (-4.31, 4.5) | 1.18 (-3.24, 5.59) | -0.22 (-4.62, 4.18) | -0.34 (-4.75, 4.07) | -0.03 (-3.85, 3.82) | -1.38 (-4.83, 2.04) | Saffloweryellow | -1.4 (-5.82, 3) | -1.08 (-5.49, 3.33) | -0.59 (-5.01, 3.83) | -7.43 (-13.79, -1.12) | 0.39 (-2.73, 3.51) | 0.3 (-4.11, 4.69) | -1.15 (-4.99, 2.67) | -0.27 (-3.88, 3.32) |  |
| Salvianolate | 1.81 (-3.56, 7.22) | 0.72 (-2.54, 3.99) | 2.08 (-1.99, 6.16) | -0.11 (-3.71, 3.48) | 0.52 (-3.51, 4.56) | -0.72 (-4.56, 3.11) | 0.63 (-3.17, 4.45) | 1.52 (-2.88, 5.93) | 2.57 (-1.82, 7) | 1.19 (-3.21, 5.6) | 1.07 (-3.32, 5.48) | 1.38 (-2.43, 5.2) | 0.03 (-3.4, 3.44) | 1.4 (-3, 5.82) | Salvianolate | 0.33 (-4.08, 4.75) | 0.82 (-3.6, 5.21) | -6.02 (-12.36, 0.31) | 1.8 (-1.32, 4.92) | 1.7 (-2.71, 6.1) | 0.25 (-3.56, 4.08) | 1.14 (-2.46, 4.73) |  |
| Shenmai | 1.48 (-3.9, 6.92) | 0.39 (-2.88, 3.66) | 1.75 (-2.34, 5.84) | -0.45 (-4.06, 3.16) | 0.19 (-3.86, 4.24) | -1.05 (-4.9, 2.79) | 0.31 (-3.51, 4.13) | 1.18 (-3.23, 5.63) | 2.25 (-2.16, 6.69) | 0.86 (-3.55, 5.3) | 0.75 (-3.68, 5.16) | 1.06 (-2.79, 4.9) | -0.31 (-3.74, 3.14) | 1.08 (-3.33, 5.49) | -0.33 (-4.75, 4.08) | Shenmai | 0.48 (-3.95, 4.92) | -6.35 (-12.71, -0.01) | 1.47 (-1.67, 4.62) | 1.37 (-3.05, 5.8) | -0.08 (-3.9, 3.77) | 0.8 (-2.81, 4.44) |  |
| Shenxiong | 0.99 (-4.38, 6.41) | -0.09 (-3.35, 3.16) | 1.27 (-2.82, 5.35) | -0.92 (-4.52, 2.66) | -0.29 (-4.34, 3.74) | -1.53 (-5.37, 2.29) | -0.18 (-3.99, 3.63) | 0.7 (-3.71, 5.11) | 1.77 (-2.64, 6.18) | 0.36 (-4.03, 4.8) | 0.26 (-4.14, 4.64) | 0.57 (-3.26, 4.38) | -0.78 (-4.21, 2.63) | 0.59 (-3.83, 5.01) | -0.82 (-5.21, 3.6) | -0.48 (-4.92, 3.95) | Shenxiong | -6.84 (-13.18, -0.52) | 0.98 (-2.13, 4.1) | 0.9 (-3.51, 5.28) | -0.56 (-4.4, 3.28) | 0.33 (-3.28, 3.92) |  |
| Shuxuetong | 7.83 (4.54, 11.13) | 6.74 (1.15, 12.31) | 8.1 (2.01, 14.19) | 5.9 (0.13, 11.69) | 6.54 (0.48, 12.63) | 5.31 (-0.68, 11.25) | 6.65 (0.74, 12.6) | 7.53 (3.04, 12.08) | 8.6 (2.28, 14.91) | 7.2 (0.88, 13.56) | 7.08 (0.77, 13.41) | 7.4 (1.49, 13.36) | 6.04 (0.38, 11.72) | 7.43 (1.12, 13.79) | 6.02 (-0.31, 12.36) | 6.35 (0.01, 12.71) | 6.84 (0.52, 13.18) | Shuxuetong | 7.81 (2.32, 13.33) | 7.72 (1.39, 14.03) | 6.27 (0.36, 12.2) | 7.16 (1.36, 12.97) |  |
| **Standard** | **0.01 (-4.39, 4.42)** | **-1.08 (-2.01, -0.15)** | **0.29 (-2.34, 2.91)** | **-1.91 (-3.71, -0.12)** | **-1.28 (-3.84, 1.28)** | **-2.51 (-4.77, -0.29)** | **-1.16 (-3.37, 1.03)** | **-0.28 (-3.4, 2.82)** | **0.78 (-2.33, 3.9)** | **-0.62 (-3.74, 2.53)** | **-0.73 (-3.85, 2.38)** | **-0.41 (-2.62, 1.81)** | **-1.77 (-3.2, -0.37)** | **-0.39 (-3.51, 2.73)** | **-1.8 (-4.92, 1.32)** | **-1.47 (-4.62, 1.67)** | **-0.98 (-4.1, 2.13)** | **-7.81 (-13.33, -2.32)** | **Standard** | **-0.09 (-3.22, 3.01)** | **-1.55 (-3.77, 0.66)** | **-0.66 (-2.47, 1.13)** |  |
| Tanshinone | 0.1 (-5.27, 5.51) | -0.99 (-4.22, 2.29) | 0.38 (-3.69, 4.46) | -1.82 (-5.39, 1.77) | -1.18 (-5.23, 2.88) | -2.42 (-6.27, 1.41) | -1.07 (-4.87, 2.75) | -0.19 (-4.57, 4.22) | 0.88 (-3.53, 5.28) | -0.52 (-4.9, 3.91) | -0.63 (-5.03, 3.77) | -0.32 (-4.13, 3.53) | -1.68 (-5.09, 1.75) | -0.3 (-4.69, 4.11) | -1.7 (-6.1, 2.71) | -1.37 (-5.8, 3.05) | -0.9 (-5.28, 3.51) | -7.72 (-14.03, -1.39) | 0.09 (-3.01, 3.22) | Tanshinone | -1.45 (-5.26, 2.37) | -0.57 (-4.17, 3.04) |  |
| Xingxiong | 1.56 (-3.37, 6.5) | 0.47 (-1.93, 2.88) | 1.83 (-1.59, 5.25) | -0.37 (-3.21, 2.48) | 0.27 (-3.12, 3.64) | -0.97 (-4.13, 2.17) | 0.39 (-2.75, 3.5) | 1.26 (-2.57, 5.09) | 2.32 (-1.49, 6.16) | 0.94 (-2.88, 4.75) | 0.82 (-3, 4.64) | 1.13 (-2, 4.28) | -0.22 (-2.85, 2.4) | 1.15 (-2.67, 4.99) | -0.25 (-4.08, 3.56) | 0.08 (-3.77, 3.9) | 0.56 (-3.28, 4.4) | -6.27 (-12.2, -0.36) | 1.55 (-0.66, 3.77) | 1.45 (-2.37, 5.26) | Xingxiong | 0.88 (-1.96, 3.74) |  |
| Xueshuantong | 0.68 (-4.1, 5.44) | -0.42 (-2.44, 1.61) | 0.94 (-2.22, 4.13) | -1.25 (-3.8, 1.3) | -0.62 (-3.75, 2.52) | -1.86 (-4.74, 1.01) | -0.5 (-3.35, 2.33) | 0.38 (-3.21, 3.98) | 1.44 (-2.15, 5.05) | 0.05 (-3.54, 3.66) | -0.07 (-3.67, 3.53) | 0.24 (-2.6, 3.11) | -1.11 (-3.4, 1.17) | 0.27 (-3.32, 3.88) | -1.14 (-4.73, 2.46) | -0.8 (-4.44, 2.81) | -0.33 (-3.92, 3.28) | -7.16 (-12.97, -1.36) | 0.66 (-1.13, 2.47) | 0.57 (-3.04, 4.17) | -0.88 (-3.74, 1.96) | Xueshuantong |  |
