# Supplementary material for: Efficacy and safety of different traditional Chinese medicine injections in the treatment of unstable angina pectoris: a systematic review and Bayesian network meta-analysis
Source: Front Pharmacol. 2025 Mar 12;16:1550759. doi: 10.3389/fphar.2025.1550759 (PMC11937076; doi:10.3389/fphar.2025.1550759)
Supplement: Supplementary file 7 [file Table4.docx]

Table S4: League table of various injections for reducing the duration of angina attacks in individuals with UAP

|  | **CompoundDS** | **Danhong** | **Danshen** | **DanshenCXQ** | **Dengzhanxixin** | **GinkgoDamole** | **GinkgoLeaf** | **Gualoupi** | **Hongjingtian** | **Kudiezi** | **LigustrazineH** | **Puerarin** | **Saffloweryellow** | **Salvianolate** | **Shenmai** | **Shenxiong** | **Shuxuetong** | **Standard** | **Tanshinone** | **Xingxiong** | **Xueshuantong** |
| --- | --- | --- | --- | --- | --- | --- | --- | --- | --- | --- | --- | --- | --- | --- | --- | --- | --- | --- | --- | --- | --- |
| CompoundDS | CompoundDS | -0.48 (-6.5, 5.48) | 1.23 (-5.61, 8.03) | -0.03 (-6.36, 6.25) | -0.45 (-7.27, 6.31) | 0.34 (-6.84, 7.5) | -1.65 (-8.2, 4.9) | -0.08 (-4.2, 4.05) | 0.22 (-6.97, 7.43) | -0.26 (-7.46, 6.89) | -0.47 (-7.01, 6.07) | -2.14 (-8.75, 4.36) | -0.01 (-7.2, 7.18) | -0.87 (-8.03, 6.3) | -1.18 (-8.39, 5.99) | 0.15 (-7.06, 7.27) | -3.6 (-7.75, 0.6) | 0.68 (-5.2, 6.53) | 0.56 (-6.64, 7.72) | -1.82 (-8.39, 4.71) | 0.03 (-6.3, 6.33) |
| Danhong | 0.48 (-5.48, 6.5) | Danhong | 1.73 (-1.69, 5.12) | 0.45 (-2.25, 3.17) | 0.03 (-3.47, 3.52) | 0.82 (-3.52, 5.17) | -1.16 (-4.35, 2.06) | 0.41 (-3.93, 4.76) | 0.71 (-3.65, 5.07) | 0.23 (-4.11, 4.59) | 0.02 (-3.19, 3.23) | -1.66 (-4.93, 1.59) | 0.49 (-3.88, 4.82) | -0.38 (-4.68, 3.98) | -0.69 (-5.05, 3.68) | 0.63 (-3.69, 5) | -3.1 (-10.38, 4.21) | 1.16 (-0.13, 2.45) | 1.04 (-3.29, 5.38) | -1.33 (-4.56, 1.87) | 0.51 (-2.2, 3.23) |
| Danshen | -1.23 (-8.03, 5.61) | -1.73 (-5.12, 1.69) | Danshen | -1.27 (-5.49, 2.95) | -1.69 (-5.1, 1.74) | -0.89 (-6.34, 4.52) | -2.88 (-7.43, 1.68) | -1.31 (-6.7, 4.1) | -1.02 (-6.46, 4.42) | -1.49 (-6.92, 3.96) | -1.7 (-6.25, 2.86) | -3.38 (-7.99, 1.19) | -1.23 (-6.67, 4.18) | -2.09 (-7.51, 3.31) | -2.42 (-7.84, 3.05) | -1.09 (-6.47, 4.32) | -4.84 (-12.8, 3.17) | -0.56 (-4.04, 2.93) | -0.67 (-6.09, 4.72) | -3.06 (-7.63, 1.49) | -1.21 (-5.43, 3.03) |
| DanshenCXQ | 0.03 (-6.25, 6.36) | -0.45 (-3.17, 2.25) | 1.27 (-2.95, 5.49) | DanshenCXQ | -0.42 (-4.6, 3.74) | 0.37 (-4.41, 5.18) | -1.61 (-5.4, 2.18) | -0.04 (-4.85, 4.74) | 0.26 (-4.55, 5.06) | -0.23 (-5.03, 4.58) | -0.44 (-4.2, 3.36) | -2.11 (-5.95, 1.71) | 0.04 (-4.73, 4.81) | -0.83 (-5.61, 3.97) | -1.15 (-5.95, 3.67) | 0.17 (-4.59, 4.97) | -3.57 (-11.1, 4.08) | 0.71 (-1.68, 3.1) | 0.59 (-4.21, 5.38) | -1.79 (-5.57, 1.98) | 0.06 (-3.31, 3.44) |
| Dengzhanxixin | 0.45 (-6.31, 7.27) | -0.03 (-3.52, 3.47) | 1.69 (-1.74, 5.1) | 0.42 (-3.74, 4.6) | Dengzhanxixin | 0.79 (-4.57, 6.14) | -1.18 (-5.71, 3.32) | 0.37 (-5, 5.76) | 0.67 (-4.69, 6.08) | 0.19 (-5.15, 5.57) | -0.01 (-4.53, 4.49) | -1.69 (-6.25, 2.83) | 0.46 (-4.93, 5.82) | -0.41 (-5.79, 4.95) | -0.73 (-6.13, 4.7) | 0.6 (-4.74, 5.97) | -3.14 (-11.09, 4.86) | 1.13 (-2.28, 4.54) | 1.01 (-4.36, 6.35) | -1.36 (-5.88, 3.15) | 0.48 (-3.69, 4.65) |
| GinkgoDamole | -0.34 (-7.5, 6.84) | -0.82 (-5.17, 3.52) | 0.89 (-4.52, 6.34) | -0.37 (-5.18, 4.41) | -0.79 (-6.14, 4.57) | GinkgoDamole | -1.98 (-7.06, 3.08) | -0.41 (-6.3, 5.43) | -0.11 (-5.98, 5.75) | -0.6 (-6.46, 5.24) | -0.81 (-5.88, 4.27) | -2.49 (-7.6, 2.61) | -0.33 (-6.19, 5.53) | -1.21 (-7.05, 4.65) | -1.52 (-7.4, 4.4) | -0.2 (-6.02, 5.65) | -3.92 (-12.23, 4.38) | 0.34 (-3.81, 4.49) | 0.21 (-5.6, 6.08) | -2.16 (-7.28, 2.92) | -0.31 (-5.06, 4.46) |
| GinkgoLeaf | 1.65 (-4.9, 8.2) | 1.16 (-2.06, 4.35) | 2.88 (-1.68, 7.43) | 1.61 (-2.18, 5.4) | 1.18 (-3.32, 5.71) | 1.98 (-3.08, 7.06) | GinkgoLeaf | 1.57 (-3.52, 6.65) | 1.87 (-3.25, 6.94) | 1.39 (-3.71, 6.45) | 1.17 (-2.97, 5.32) | -0.5 (-4.72, 3.68) | 1.64 (-3.45, 6.71) | 0.78 (-4.29, 5.85) | 0.46 (-4.63, 5.57) | 1.79 (-3.27, 6.87) | -1.95 (-9.7, 5.85) | 2.32 (-0.61, 5.25) | 2.2 (-2.88, 7.27) | -0.18 (-4.37, 3.97) | 1.67 (-2.1, 5.46) |
| Gualoupi | 0.08 (-4.05, 4.2) | -0.41 (-4.76, 3.93) | 1.31 (-4.1, 6.7) | 0.04 (-4.74, 4.85) | -0.37 (-5.76, 5) | 0.41 (-5.43, 6.3) | -1.57 (-6.65, 3.52) | Gualoupi | 0.3 (-5.54, 6.17) | -0.19 (-6.02, 5.68) | -0.39 (-5.47, 4.67) | -2.07 (-7.21, 3.03) | 0.07 (-5.81, 5.96) | -0.79 (-6.63, 5.1) | -1.1 (-6.96, 4.78) | 0.23 (-5.63, 6.08) | -3.52 (-9.39, 2.38) | 0.75 (-3.39, 4.9) | 0.64 (-5.25, 6.51) | -1.74 (-6.85, 3.35) | 0.11 (-4.69, 4.91) |
| Hongjingtian | -0.22 (-7.43, 6.97) | -0.71 (-5.07, 3.65) | 1.02 (-4.42, 6.46) | -0.26 (-5.06, 4.55) | -0.67 (-6.08, 4.69) | 0.11 (-5.75, 5.98) | -1.87 (-6.94, 3.25) | -0.3 (-6.17, 5.54) | Hongjingtian | -0.49 (-6.35, 5.41) | -0.69 (-5.78, 4.43) | -2.37 (-7.5, 2.72) | -0.22 (-6.12, 5.66) | -1.08 (-6.98, 4.79) | -1.4 (-7.3, 4.49) | -0.08 (-5.96, 5.78) | -3.81 (-12.11, 4.51) | 0.45 (-3.71, 4.62) | 0.33 (-5.54, 6.22) | -2.04 (-7.15, 3.05) | -0.18 (-5, 4.59) |
| Kudiezi | 0.26 (-6.89, 7.46) | -0.23 (-4.59, 4.11) | 1.49 (-3.96, 6.92) | 0.23 (-4.58, 5.03) | -0.19 (-5.57, 5.15) | 0.6 (-5.24, 6.46) | -1.39 (-6.45, 3.71) | 0.19 (-5.68, 6.02) | 0.49 (-5.41, 6.35) | Kudiezi | -0.21 (-5.3, 4.88) | -1.88 (-7, 3.19) | 0.26 (-5.63, 6.11) | -0.6 (-6.46, 5.25) | -0.91 (-6.77, 4.98) | 0.41 (-5.46, 6.24) | -3.33 (-11.58, 4.97) | 0.93 (-3.2, 5.06) | 0.81 (-5.05, 6.68) | -1.56 (-6.68, 3.49) | 0.29 (-4.49, 5.09) |
| LigustrazineH | 0.47 (-6.07, 7.01) | -0.02 (-3.23, 3.19) | 1.7 (-2.86, 6.25) | 0.44 (-3.36, 4.2) | 0.01 (-4.49, 4.53) | 0.81 (-4.27, 5.88) | -1.17 (-5.32, 2.97) | 0.39 (-4.67, 5.47) | 0.69 (-4.43, 5.78) | 0.21 (-4.88, 5.3) | LigustrazineH | -1.68 (-5.86, 2.51) | 0.47 (-4.63, 5.55) | -0.39 (-5.46, 4.7) | -0.7 (-5.8, 4.39) | 0.61 (-4.44, 5.7) | -3.13 (-10.86, 4.69) | 1.14 (-1.78, 4.09) | 1.02 (-4, 6.12) | -1.36 (-5.52, 2.81) | 0.5 (-3.28, 4.29) |
| Puerarin | 2.14 (-4.36, 8.75) | 1.66 (-1.59, 4.93) | 3.38 (-1.19, 7.99) | 2.11 (-1.71, 5.95) | 1.69 (-2.83, 6.25) | 2.49 (-2.61, 7.6) | 0.5 (-3.68, 4.72) | 2.07 (-3.03, 7.21) | 2.37 (-2.72, 7.5) | 1.88 (-3.19, 7) | 1.68 (-2.51, 5.86) | Puerarin | 2.14 (-2.94, 7.29) | 1.28 (-3.79, 6.41) | 0.96 (-4.16, 6.13) | 2.28 (-2.8, 7.42) | -1.44 (-9.15, 6.38) | 2.82 (-0.14, 5.81) | 2.7 (-2.36, 7.83) | 0.32 (-3.86, 4.53) | 2.17 (-1.6, 6.02) |
| Saffloweryellow | 0.01 (-7.18, 7.2) | -0.49 (-4.82, 3.88) | 1.23 (-4.18, 6.67) | -0.04 (-4.81, 4.73) | -0.46 (-5.82, 4.93) | 0.33 (-5.53, 6.19) | -1.64 (-6.71, 3.45) | -0.07 (-5.96, 5.81) | 0.22 (-5.66, 6.12) | -0.26 (-6.11, 5.63) | -0.47 (-5.55, 4.63) | -2.14 (-7.29, 2.94) | Saffloweryellow | -0.86 (-6.7, 4.98) | -1.17 (-7.04, 4.73) | 0.14 (-5.71, 6.03) | -3.59 (-11.87, 4.76) | 0.68 (-3.46, 4.84) | 0.56 (-5.31, 6.44) | -1.82 (-6.91, 3.27) | 0.03 (-4.75, 4.84) |
| Salvianolate | 0.87 (-6.3, 8.03) | 0.38 (-3.98, 4.68) | 2.09 (-3.31, 7.51) | 0.83 (-3.97, 5.61) | 0.41 (-4.95, 5.79) | 1.21 (-4.65, 7.05) | -0.78 (-5.85, 4.29) | 0.79 (-5.1, 6.63) | 1.08 (-4.79, 6.98) | 0.6 (-5.25, 6.46) | 0.39 (-4.7, 5.46) | -1.28 (-6.41, 3.79) | 0.86 (-4.98, 6.7) | Salvianolate | -0.31 (-6.19, 5.56) | 1.01 (-4.84, 6.86) | -2.73 (-11.02, 5.6) | 1.55 (-2.59, 5.66) | 1.43 (-4.44, 7.29) | -0.96 (-6.04, 4.1) | 0.89 (-3.86, 5.67) |
| Shenmai | 1.18 (-5.99, 8.39) | 0.69 (-3.68, 5.05) | 2.42 (-3.05, 7.84) | 1.15 (-3.67, 5.95) | 0.73 (-4.7, 6.13) | 1.52 (-4.4, 7.4) | -0.46 (-5.57, 4.63) | 1.1 (-4.78, 6.96) | 1.4 (-4.49, 7.3) | 0.91 (-4.98, 6.77) | 0.7 (-4.39, 5.8) | -0.96 (-6.13, 4.16) | 1.17 (-4.73, 7.04) | 0.31 (-5.56, 6.19) | Shenmai | 1.32 (-4.56, 7.19) | -2.41 (-10.73, 5.91) | 1.85 (-2.32, 6.02) | 1.73 (-4.12, 7.61) | -0.64 (-5.77, 4.46) | 1.21 (-3.61, 6) |
| Shenxiong | -0.15 (-7.27, 7.06) | -0.63 (-5, 3.69) | 1.09 (-4.32, 6.47) | -0.17 (-4.97, 4.59) | -0.6 (-5.97, 4.74) | 0.2 (-5.65, 6.02) | -1.79 (-6.87, 3.27) | -0.23 (-6.08, 5.63) | 0.08 (-5.78, 5.96) | -0.41 (-6.24, 5.46) | -0.61 (-5.7, 4.44) | -2.28 (-7.42, 2.8) | -0.14 (-6.03, 5.71) | -1.01 (-6.86, 4.84) | -1.32 (-7.19, 4.56) | Shenxiong | -3.75 (-12, 4.64) | 0.53 (-3.62, 4.66) | 0.41 (-5.43, 6.26) | -1.96 (-7.08, 3.12) | -0.11 (-4.9, 4.67) |
| Shuxuetong | 3.6 (-0.6, 7.75) | 3.1 (-4.21, 10.38) | 4.84 (-3.17, 12.8) | 3.57 (-4.08, 11.1) | 3.14 (-4.86, 11.09) | 3.92 (-4.38, 12.23) | 1.95 (-5.85, 9.7) | 3.52 (-2.38, 9.39) | 3.81 (-4.51, 12.11) | 3.33 (-4.97, 11.58) | 3.13 (-4.69, 10.86) | 1.44 (-6.38, 9.15) | 3.59 (-4.76, 11.87) | 2.73 (-5.6, 11.02) | 2.41 (-5.91, 10.73) | 3.75 (-4.64, 12) | Shuxuetong | 4.27 (-2.95, 11.44) | 4.14 (-4.19, 12.44) | 1.78 (-6.04, 9.5) | 3.62 (-3.99, 11.16) |
| **Standard** | **-0.68 (-6.53, 5.2)** | **-1.16 (-2.45, 0.13)** | **0.56 (-2.93, 4.04)** | **-0.71 (-3.1, 1.68)** | **-1.13 (-4.54, 2.28)** | **-0.34 (-4.49, 3.81)** | **-2.32 (-5.25, 0.61)** | **-0.75 (-4.9, 3.39)** | **-0.45 (-4.62, 3.71)** | **-0.93 (-5.06, 3.2)** | **-1.14 (-4.09, 1.78)** | **-2.82 (-5.81, 0.14)** | **-0.68 (-4.84, 3.46)** | **-1.55 (-5.66, 2.59)** | **-1.85 (-6.02, 2.32)** | **-0.53 (-4.66, 3.62)** | **-4.27 (-11.44, 2.95)** | **Standard** | **-0.12 (-4.26, 4.01)** | **-2.5 (-5.46, 0.43)** | **-0.65 (-3.03, 1.75)** |
| Tanshinone | -0.56 (-7.72, 6.64) | -1.04 (-5.38, 3.29) | 0.67 (-4.72, 6.09) | -0.59 (-5.38, 4.21) | -1.01 (-6.35, 4.36) | -0.21 (-6.08, 5.6) | -2.2 (-7.27, 2.88) | -0.64 (-6.51, 5.25) | -0.33 (-6.22, 5.54) | -0.81 (-6.68, 5.05) | -1.02 (-6.12, 4) | -2.7 (-7.83, 2.36) | -0.56 (-6.44, 5.31) | -1.43 (-7.29, 4.44) | -1.73 (-7.61, 4.12) | -0.41 (-6.26, 5.43) | -4.14 (-12.44, 4.19) | 0.12 (-4.01, 4.26) | Tanshinone | -2.37 (-7.48, 2.68) | -0.52 (-5.32, 4.25) |
| Xingxiong | 1.82 (-4.71, 8.39) | 1.33 (-1.87, 4.56) | 3.06 (-1.49, 7.63) | 1.79 (-1.98, 5.57) | 1.36 (-3.15, 5.88) | 2.16 (-2.92, 7.28) | 0.18 (-3.97, 4.37) | 1.74 (-3.35, 6.85) | 2.04 (-3.05, 7.15) | 1.56 (-3.49, 6.68) | 1.36 (-2.81, 5.52) | -0.32 (-4.53, 3.86) | 1.82 (-3.27, 6.91) | 0.96 (-4.1, 6.04) | 0.64 (-4.46, 5.77) | 1.96 (-3.12, 7.08) | -1.78 (-9.5, 6.04) | 2.5 (-0.43, 5.46) | 2.37 (-2.68, 7.48) | Xingxiong | 1.84 (-1.93, 5.67) |
| Xueshuantong | -0.03 (-6.33, 6.3) | -0.51 (-3.23, 2.2) | 1.21 (-3.03, 5.43) | -0.06 (-3.44, 3.31) | -0.48 (-4.65, 3.69) | 0.31 (-4.46, 5.06) | -1.67 (-5.46, 2.1) | -0.11 (-4.91, 4.69) | 0.18 (-4.59, 5) | -0.29 (-5.09, 4.49) | -0.5 (-4.29, 3.28) | -2.17 (-6.02, 1.6) | -0.03 (-4.84, 4.75) | -0.89 (-5.67, 3.86) | -1.21 (-6, 3.61) | 0.11 (-4.67, 4.9) | -3.62 (-11.16, 3.99) | 0.65 (-1.75, 3.03) | 0.52 (-4.25, 5.32) | -1.84 (-5.67, 1.93) | Xueshuantong |
